# Best practices for germline variant and DNA methylation analysis of second- and third-generation sequencing data

**DOI:** 10.1186/s40246-024-00684-8

**Published:** 2024-11-05

**Authors:** Ferdinando Bonfiglio, Andrea Legati, Vito Alessandro Lasorsa, Flavia Palombo, Giulia De Riso, Federica Isidori, Silvia Russo, Simone Furini, Giuseppe Merla, Fabio Coppedè, Marco Tartaglia, Omics Sciences - Bioinformatics and Epigenetics Working Groups of the Italian Society of Human Genetics (SIGU), Alessandro Bruselles, Tommaso Pippucci, Andrea Ciolfi, Michele Pinelli, Mario Capasso

**Affiliations:** 1 Department of Molecular Medicine and Medical Biotechnology, University of Naples Federico II, Naples, Italy; 2 CEINGE Advanced Biotechnology Franco Salvatore, Naples, Italy; 3 Fondazione IRCCS Istituto Neurologico Carlo Besta, Milan, Italy; 4 Programma Di Neurogenetica, IRCCS Istituto Delle Scienze Neurologiche Di Bologna, Bologna, Italy; 5 IRCCS Azienda Ospedaliero-Universitaria Di Bologna, Bologna, Italy; 6 Research Laboratory of Medical Cytogenetics and Molecular Genetics, IRCCS Istituto Auxologico Italiano, Milan, Italy; 7 Department of Electrical, Electronic and Information Engineering “Guglielmo Marconi”, University of Bologna, Bologna, Italy; 8 Department of Translational Research and of New Surgical and Medical Technologies, University of Pisa, Pisa, Italy; 9 Molecular Genetics and Functional Genomics, Bambino Gesù Children’s Hospital, IRCCS, Rome, Italy; 10 Department of Oncology and Molecular Medicine, Istituto Superiore Di Sanità, Rome, Italy; 11 Laboratorio di Ricerca di Citogenetica Medica e Genetica Molecolare, Istituto Auxologico Italiano, IRCCS, 20145 Milano, Italy

**Keywords:** Germline variants, DNA methylation, NGS, Hereditary diseases, Bioinformatics, Genetic diagnostics

## Abstract

This comprehensive review provides insights and suggested strategies for the analysis of germline variants using second- and third-generation sequencing technologies (SGS and TGS). It addresses the critical stages of data processing, starting from alignment and preprocessing to quality control, variant calling, and the removal of artifacts. The document emphasized the importance of meticulous data handling, highlighting advanced methodologies for annotating variants and identifying structural variations and methylated DNA sites. Special attention is given to the inspection of problematic variants, a step that is crucial for ensuring the accuracy of the analysis, particularly in clinical settings where genetic diagnostics can inform patient care. Additionally, the document covers the use of various bioinformatics tools and software that enhance the precision and reliability of these analyses. It outlines best practices for the annotation of variants, including considerations for problematic genetic alterations such as those in the human leukocyte antigen region, runs of homozygosity, and mitochondrial DNA alterations. The document also explores the complexities associated with identifying structural variants and copy number variations, underscoring the challenges posed by these large-scale genomic alterations. The objective is to offer a comprehensive framework for researchers and clinicians, ensuring that genetic analyses conducted with SGS and TGS are both accurate and reproducible. By following these best practices, the document aims to increase the diagnostic accuracy for hereditary diseases, facilitating early diagnosis, prevention, and personalized treatment strategies. This review serves as a valuable resource for both novices and experts in the field, providing insights into the latest advancements and methodologies in genetic analysis. It also aims to encourage the adoption of these practices in diverse research and clinical contexts, promoting consistency and reliability across studies.

## Introduction

Over the last 20 years, sequencing advances have significantly surpassed traditional Sanger sequencing methods, ushering in the era of "next- or second-generation sequencing" (SGS), which allows for the simultaneous sequencing of millions to billions of short sequences in parallel. However, rapid technological innovations are already bringing us into the third era of sequencing, where long-read technologies enable the sequencing of very cryptic genomic regions.

The analysis of germline variants via SGS or third-generation sequencing (TGS) represents a crucial field in human genetics and molecular medicine. These variants can significantly impact diagnosis and susceptibility to hereditary diseases and influence responses to medical treatments. Therefore, their accurate identification is essential for early diagnosis, prevention, and management of genetic diseases, particularly rare ones.

SGS and TGS have revolutionized the ability to detect and characterize germline variants effectively at the genome level. SGS enables the parallel reading of millions of DNA fragments, allowing for high coverage and precise data generation. Furthermore, TGS, such as the PacBio system and Nanopore technologies, now offers the possibility of real-time reading of much longer DNA fragments, providing more comprehensive information on gene structure and variants.

However, the analysis of germline variants using these technologies requires rigorous adherence to guidelines and best practices to ensure reliable results. This includes careful preparation of the sample, the sequencing process itself, and the subsequent data analysis stages. Errors in variant calling can have severe consequences, leading to incorrect diagnoses or inadequate therapeutic decisions.

This document describes and discusses the various processes applied to high-throughput sequencing data analysis with the intent of providing key best practices for germline variant analysis via SGS and TGS. It also describes different genome-wide approaches to evaluate methylated DNA (DNAm) levels. The focus is exclusively on germline variations, as the characterization of somatic variations is beyond the scope of this review. For those seeking to deepen their understanding of the clinical application of SGS technologies, we recommend referring to the comprehensive guidelines developed by EuroGentest and the European Society of Human Genetics [1]. These guidelines offer essential insights into the implementation, validation, and accreditation of SGS in clinical laboratories. The document provides practical recommendations, including quality assurance measures and a structured framework for integrating NGS into diagnostic workflows, ensuring accuracy, reliability, and standardization across laboratories.

### Aim

The main objective is to review the various analytical tools used in the different stages of computational data analysis obtained from SGS and TGS, with a focus on the genome-wide calling of small and large germline variants, as well as those not commonly considered, such as human leukocyte antigen (HLA) genotypes, runs of homozygosity (ROH), and mitochondrial DNA (mtDNA) alterations. Targeted and comprehensive genomic methods for the identification of DNAm sites will also be explored, with a specific focus on the diagnosis and research of genetic diseases.

This document is intended for a diverse audience ranging from beginners in the field to experts interested in delving into innovative topics such as third-generation data analysis and less-studied alterations related to HLA, ROH, mtDNA, and DNAm. The intent is to provide interesting and stimulating insights that can attract attention and encourage further exploration.

### Methodology

The recommendations presented in this document are based on a comprehensive review of relevant literature, including peer-reviewed original articles, review articles, guidelines, comparative studies, case reports, methodological articles, as well as perspective and opinion papers. Priority was giving to the analysis of original articles, review articles, guidelines, and comparative studies. Grey literature, manufacturers' datasheets, and personal communications were not included. Abstracts from recent meetings (2022–2024) were also considered. Documents were selected based on their relevance to SGS, TGS and genome-wide methylation applications, as well as their reliability and significance in both clinical and research settings. Key information was extracted from these sources to inform the recommendations and strategies outlined in this document.

## Second-generation sequencing

The evolution of computational analyses has had a revolutionary impact on the application of SGS in the diagnosis and research of rare genetic diseases. These techniques have significantly improved the ability to detect, characterize, and interpret genetic variants, transforming how rare diseases are studied and diagnosed. Before the advent of SGS and related computational developments, identifying the genetic basis of rare diseases was a long, costly, and often unsuccessful process. However, with SGS, it is now possible to sequence entire genomes or exomes rapidly and affordably, generating an enormous amount of data.

Computational analyses play a critical role in processing these data, from aligning sequences to the reference genome to variant calling and annotation and interpretation. Advanced software and dedicated algorithms enable effective artifact filtering, identification of pathogenic variants, and identification of associations with specific genetic conditions, increasing the speed, accuracy, and accessibility of the diagnosis of rare diseases. These techniques have also opened new frontiers in research, facilitating the discovery of previously unknown genes associated with rare diseases and unveiling the molecular mechanisms underlying these conditions.

### Overview of different SGS platforms

Several companies have developed different technologies with unique strengths. Illumina, Element Biosciences, Ultima Genomics, ThermoFisher (Ion Torrent) and MGI represent distinct approaches with varying technologies and applications. Illumina, the market leader, uses sequencing by synthesis, offering high accuracy with shorter read lengths and broad applications, particularly in clinical and research genomics. Platforms like NovaSeq provide supports whole genome sequencing (WGS) of humans, plants, and animals [2]. Element Biosciences' Aviti platform also employs sequencing by synthesis but focuses on reducing sequencing costs while maintaining comparable accuracy to Illumina. Studies show that Aviti can generate cleaner data with fewer false positives than Illumina’s systems [3]. Ultima Genomics is a newer, disruptive platform focused on ultra-low-cost sequencing, aiming to reduce the cost of WGS to $100. However, the specifics of its novel approach are proprietary and still undergoing broader adoption. Ion Torrent is a SGS platform that uses a unique technology called semiconductor sequencing. Unlike other platforms, Ion Torrent directly detects hydrogen ions (protons) released during nucleotide incorporation. This process allows for real-time, label-free sequencing, making the Ion Torrent system faster and less expensive compared to some other platforms. genomic filed [2]. Finally, MGI (a subsidiary of BGI) uses *DNBSEQ technology*, offering DNA nanoball-based sequencing that is known for high accuracy and low costs, comparable to Illumina but with unique features like reduced duplication rates [4]. A study demonstrated that the sequencing throughput and turnaround time, single-base quality, read quality, and variant calling were similar to Illumina HiSeq2500 data [5].

### Alignment and preprocessing

This section focuses on the importance of correct alignment and preprocessing of data in the SGS field, especially in the context of mendelian diseases. These preliminary stages are fundamental to ensuring that the sequencing data analysis is accurate and meaningful. Through alignment, sequenced DNA fragments are correctly positioned on the reference genomic sequence of the studied organism, allowing for the identification of relevant genetic variations. Preprocessing, on the other hand, involves cleaning and normalizing the data to remove artifacts and experimental biases, thus improving the quality and reliability of the results.

#### Quality control

Illumina sequencing involves transforming original fluorescence signals into "reads" (short nucleotide sequences obtained from the sequencing process) during the base-calling phase. Reads are saved in text files in a standard format called FASTQ (.fq or .fastq). Each read is represented by the nucleotide sequence of the DNA fragment from which it derives and by the quality values of each nucleotide (reported in the Phred logarithmic scale as the probability of a reading error).

Quality control (QC) and preprocessing of FASTQ files are essential to ensure the reliability of downstream analyses, such as variant calling. Typically, QC involves the following:

1. Recognizing and removing any sequencing adapters;
2. Recognizing and removing any reads containing undetermined nucleotides ("N") for More than 10% of their length;
3. Recognizing and removing any reads containing low-quality nucleotides (usually Q_PHRED_ < 5 for more than 50% of their length).

The Q_PHRED_ (Phred quality) score is a metric used in SGS to estimate the quality of base calls. It reflects the likelihood that a base is incorrectly identified.

The score is calculated using a logarithmic scale, where a Q_PHRED_ scores of 20 and 30 indicate a 1% and 0.1% probability of erroneous base call. Indeed, the higher the score, the higher the accuracy of the base call. Achieving Q40 significantly benefits applications requiring high precision, such as clinical diagnostics, rare variant detection, and large-scale population genomics, where even minor inaccuracies could lead to significant errors in data interpretation.

In accordance with the most commonly used approaches, different software is required for each operation, such as initial quality control, adapter trimming, quality filtering, and final quality control. The most widely used software for quality control and the concurrent collection of descriptive metrics on FASTQ files is FastQC [6], which also includes a graphical interface. For adapter trimming and low-quality read removal, the most commonly used tools are Cutadapt and Trimmomatic [7, 8].

As the number of sequenced samples, sequencing yields, and read lengths increase, these multistep approaches are becoming less applicable because they require continuous user verification and numerous steps of reading/writing files, making this phase slow and inefficient. Recently, programs such as AfterQC[9] and fastp[10] have been developed to integrate all necessary steps into a single analysis. Among these, fastp has become one of the most widely used programs in the quality control phase because of its rapid execution.

#### Reference genome

The alignment phase (detailed in the next section) requires the sequencing reads and the reference genomic sequence of the studied organism. The reference genome of an organism (reference assembly) is represented by consensus sequences assembled (called contigs) to reproduce the sequences of various chromosomes as faithfully as possible (some chromosomal regions are difficult to assemble or locate).

Although the *Homo sapiens* genome is the best characterized and best known, many efforts are still being made to obtain a complete (gap-free) version that can represent the genetic diversity of different human populations. For humans and other model organisms, reference assemblies are curated and released by the Genome Reference Consortium (GRC).

Currently, the most widely used versions of the human reference genome are hg19 (GRCh37, 2009) and hg38 (GRCh38, 2013). Additionally, the recent publication of new assemblies by the Telomere-to-Telomere Consortium (T2T, January 2022) and the Human Pangenome Reference Consortium is noteworthy. Primary versions of the assemblies, therefore, report the sequences of the canonical chromosomes (1–22, X, Y for humans), the mitochondrial chromosome, and various unplaced and/or unlocalized contigs.

An uncareful choice of the reference assembly version will impact the results of downstream phases; therefore, its selection must be considered in advance on the basis of the study purposes. For example, including alternative haplotypes of hypervariable regions, such as the major histocompatibility complex (MHC) locus or the pseudoautosomal regions of chromosome Y, may result in the loss of unique mapping for some genes and thus reduce variant identification sensitivity. On the other hand, including unplaced and unlocalized contigs prevents erroneous mapping of reads originating from these genomic regions and avoids many false-positive calls.

The general recommendation is to use the so-called primary versions of the assemblies (see above), unless a specific study objective requires the use of an extended or more reduced version [11]. After the files containing the reference genome sequences are downloaded (in FASTA format), indexing is essential. This step is critical in optimizing and accelerating the reading of the genome sequence via alignment software. Importantly, each alignment software requires the index file to be in a specific format. The indexing phase is necessary only once, unless a different reference assembly is selected or the mapping software is changed.

Moreover, the choice of the reference genome version has implications for subsequent variant annotation phases. Indeed, variants must be annotated using databases developed from the same reference genome version to ensure consistency and accuracy in the interpretation of the results.

#### Stages of alignment

SGS produces many short reads (100–200 bases) for each whole-exome sequencing (WES) experiment, often reaching tens of millions. These reads are stored in FASTQ files. Alignment (or mapping) is the process by which the sequence of each read is compared to the reference genome of the studied organism. The main goal is to identify the precise genomic region (including the chromosome, start, and end positions) from which each read originates. During this comparison, every mismatch between the reads and reference sequence is also recorded.

The results of these alignments are commonly stored in BAM (.bam) files, which have become the standard for managing, storing, manipulating, and sharing alignment data. This type of file is also the starting point for various downstream analyses, including variant calling, which can involve single nucleotide variants (SNVs), copy number variants (CNVs), or structural variants (SVs).

In general, immediately after the alignment phase, the BAM file undergoes several processing steps, including the following:

- **Sorting**: Aligned reads are ordered on the basis of their genomic coordinates, facilitating subsequent analyses and improving data access efficiency.
- **Marking PCR duplicates**: Redundant reads derived from the same DNA molecule are identified and marked. These duplicate reads are generally excluded from downstream analyses to prevent data distortion.
- **Indexing**: An index file (.bai format) is created, allowing rapid and efficient programmatic access to the BAM file. This index file is crucial for numerous tools used in subsequent phases, such as variant calling, postalignment quality control, and alignment visualization, through software such as the Integrative Genomics Viewer (IGV) [12].

GATK [13], one of the most widely used software programs for variant calling, recommends two additional preprocessing steps for BAM files before proceeding with variant calling [13]. The first step is the recalibration of the original base quality scores calculated from the primary sequencing data (BQSR—base quality score recalibration), whereas the second step involves local realignment around insertions and deletions (InDels) to minimize false-positive variants caused by alignment artifacts. Although these procedures can lead to improvements, the benefits are often marginal and associated with significant computational burdens [14]. Therefore, implementing these two additional steps in the analysis can be considered optional [15].

Before performing downstream analyses, it is crucial to conduct quality control of the processed BAM files to evaluate essential metrics that ensure the reliability of the results. Sequencing-related metrics include the percentage of PCR duplicates, read coverage over sequenced regions, average sequencing depth, and percentage of sequenced regions covered by a minimum number of reads, such as 10 or 20, depending on specific depth and coverage requirements. Other important alignment-derived indicators include the percentage of mapped reads, uniquely mapped reads, and reads mapped at high-quality levels.

#### Alignment tools

Alignments can represent a bottleneck in SGS analyses because of the ever-increasing volume of sequencing data and the time required for processing. Therefore, the continuous development of new mapping tools often seeks to balance accuracy and speed. An exhaustive comparison of the most recent alignment software was carried out by Donato et al. [16].

Many programs for SGS data alignment (DNA-Seq in this case) are open source (freely licensed). Among these, the most widely used software programs are BWA (and specifically its algorithm BWA-Mem) and Bowtie2 [17, 18]. Owing to its high accuracy and execution speed, BWA-Mem is currently the most widely used alignment software for SGS data. Notably, recent developments in the BWA-Mem2 program have produced identical results in half the execution time, and BWA-Meme, which further reduces the execution time compared with BWA-Mem2, still delivers identical results [19, 20].

#### Post-alignment manipulation and quality control tools

SAMtools software is typically the preferred tool for processing raw BAM files [21]. This software includes functions for sorting, marking PCR duplicates, and indexing BAM files. The same software can be used to collect descriptive metrics useful for quality control of the final BAM. Numerous programs have been developed for BAM file manipulation and quality control. Among these tools are Picard tools (broadinstitute.github.io/picard) and GATK [13].

Recently developed Biobambam2 [22] can integrate sorting, marking PCR duplicates, and indexing into a single step, significantly accelerating the creation of the final BAM ready for subsequent analyses.

#### Key points summary

SGS has revolutionized the diagnosis and the study of rare genetic diseases by enabling the discovery of causative variants through rapid and affordable genome or exome sequencing. The improvements of SGS platforms and their technologies are enhancing the sequencing accuracy and yields while reducing costs. Computational tools play a critical role in analyzing this enormous data, improving the detection and interpretation of genetic variants. Proper alignment and preprocessing, including quality control and the use of reference genomes, are essential for reliable analysis. Tools like BWA-Mem, FastQC, and SAMtools play key roles in ensuring data accuracy, while preprocessing steps like duplicate marking and recalibration enhance variant detection. Continuous improvements in alignment and quality control tools help streamline the growing complexity of sequencing data analysis.

## SNV/InDel variant calling

Variant calling is a fundamental step in SGS analysis and is crucial for identifying genetic variations compared with the reference genome. This process is particularly relevant in biomedical research and clinical diagnostics, especially in the diagnosis of rare genetic diseases. Genetic variants are typically classified into three main categories: SNVs, small InDels (typically defined as 2–50 bp), and larger structural variants (SVs, typically defined as > 1 kb). This section focuses on the calling of germline SNVs and InDels.

### Analysis stages

The input for variant calling programs is typically a BAM file resulting from mapping, possibly processed through duplicate marking and base quality recalibration. Variant calling is performed via a probabilistic model to distinguish between experimental reading errors and true differences from the reference genome. This phase is usually followed by a filtering stage aimed at reducing the number of false positives. Various methods exist for filtering variants, ranging from predefined quality parameter thresholds to applying machine learning methods. The standard output format is the Variant Call Format (VCF) or genomic VCF (GVCF). Both formats organize information by genomic position, with each row corresponding to a position, listing the reference sequence, all observed alternative alleles, and experimental or bioinformatic algorithm results for each analyzed sample. The difference between VCF and GVCF is that the former lists only positions with differences from the reference genome, whereas the latter reports all sequenced positions, and it is better suited to perform joint analysis of a cohort in subsequent steps.

### Tools for SNV/InDel variant calling

Over the years, numerous algorithms for SNV and InDel variant calling have been proposed. These algorithms can be divided into two main types: those based on a probabilistic error model and those using data-driven machine learning methods. In the first case, the error model estimates the probabilities of different genotypes. Among the most commonly used algorithms employing this strategy is GATK-HaplotypeCaller [23]. The process used by GATK-HaplotypeCaller involves four stages:

1. Identifying regions with the highest probability of containing variants;
2. Identifying haplotypes;
3. Estimating haplotype probabilities given the reads;
4. Estimating posterior genotype probabilities.

At the end of these operations, a VCF or GVCF file is produced for each sample. It is possible to combine the calls of multiple samples in a joint call using individual GVCF files as input. Variant calling with GATK-HaplotypeCaller is usually followed by a filtering stage to reduce the number of false positives. Filtering can be performed by applying thresholds to the quality values of called variants or, more commonly, using Gaussian mixture models or machine learning models based on convolutional neural networks. The entire variant calling and subsequent filtering procedure is described in GATK guidelines.

The most commonly used machine learning-based algorithm is DeepVariant [24]. DeepVariant involves an initial phase to determine a set of possible variants with a permissive approach similar to GATK-HaplotypeCaller steps (1) and (2). For each identified variant, a tensor encoding information on bases present in reads, base qualities, mapping qualities, strand information, whether the read supports the variant or reference, and the presence of other differences from the reference in reads is defined. This information is input into a convolutional neural network trained with Genome In a Bottle (GIAB) data, a consortium that develops genomic references for validating genetic variants. The main difference between the GATK and DeepVariant approaches is that the latter does not require assumptions about the error model. Similar to other machine learning models, a sufficiently large training dataset is required for its effective application.

Several comparative analyses of variant calling programs are available in the literature (Table 1), with one of the most comprehensive and recent programs at the time of writing reported by Barbitoff et al. [25].

**Table 1:** Tools for SNV and InDel variant calling

Tool	Version	Year	Input	Output	Link
DEEPVARIANT	1.6.0 (10/2023)	2018	BAM	VCF	https://github.com/google/deepvariant
STRELKA2	2.9.10 (11/2018)	2018	BAM	VCF	https://github.com/Illumina/strelka
GATK4	4.5.0 (12/2023)	2018	BAM	VCF	https://gatk.broadinstitute.org/hc/en-us
CLAIR3	1.0.5 (12/2023)	2022	BAM	VCF	https://github.com/HKU-BAL/Clair3
OCTOPUS	0.7.4 (05/2021)	2021	BAM	VCF	https://github.com/luntergroup/octopus

The accuracy of variant calling tools is usually evaluated via comparisons with gold-standard variants provided by the GIAB consortium. Given that GIAB samples are typically used for training machine learning algorithms used for variant calling/filtering, the comparison might not accurately reveal overfitting. Nonetheless, DeepVariant currently appears to have the best performance for both genome and exome data. Similar performances are reported for Clair3, Strelka2, and Octopus. The accuracy of the variant calling procedure is strongly influenced by the filtering stage. In particular, for GATK and Octopus, filtering with convolutional neural networks and random forests, respectively, leads to a significant drop in sensitivity in exome analyses. This decrease in sensitivity is due primarily to the filtering of InDels near coding region boundaries and does not significantly impact SNVs. These comparisons are essential for understanding the strengths and weaknesses of each tool, guiding the appropriate choice depending on the analysis scenario.

### Key points summary

Variant calling is a critical process in SGS for identifying genetic variations like SNVs and small InDels, particularly important in diagnosing rare genetic diseases. The process uses BAM files and probabilistic models to distinguish between sequencing errors and true genetic variants, often followed by filtering to reduce false positives. GATK-HaplotypeCaller and DeepVariant are commonly used for this process, along with other tools that offer similar performance, such as Clair3, Strelka2, and Octopus. Filtering can reduce sensitivity for InDels near coding regions, but has less effect on SNVs. These tools and strategies help refine the identification of genetic variants and guide their appropriate use in different scenarios.

## Variant filtering to remove artifacts

Sequencing artifacts are variations introduced by non-biological processes during SGS. For example, the presence of SNVs or InDels observed in sequencing data does not origin from the original biological samples. These artifacts are often difficult to distinguish from real variants, increasing the risk of false-positive and false-negative variant calls. Identifying whether a variant is real, or an artifact is crucial, especially in clinical contexts.

### Origin of artifacts

Artifacts can arise from various stages of the SGS process, including library preparation. For example, DNA damage caused by formalin and paraffin treatments can create artifacts, which can result in excessive DNA fragmentation due to prolonged storage [26]. Exposure to oxidation products such as 8-oxoG can also introduce artifactual variations [27].

PCR represents another significant source of artifacts. Problems such as incorrect incorporations or template switching, as well as biases in the representation of specific cell populations, can occur. Approximately 0.1–1% of bases may be erroneously identified due to errors in PCR cycles, cluster amplification, sequencing cycles, and image analysis.

Library preparation kits can influence sequencing quality. For example, compared with Agilent SureSelect kits, HyperPlus kits tend to generate SNV and InDel artifacts [28].

Variant calling software can also produce artifacts that are often related to alignment errors [14]. However, many of these artifacts can be systematically filtered via methods such as the frequency hard filter [29] and VQSR [13, 30]. These methods use different strategies to optimize filtering, but the choice of the most suitable method may depend on the specific variant calling software used. However, visually inspecting alignments for clinically relevant variants via tools such as Integrative IGV [12] to identify false-positive variant calls that may escape automatic filters is recommended.

### Common types of artifacts

- **Low-quality nucleotides in multiple reads**: calls due to low-quality nucleotides in multiple reads (see alignment and preprocessing – quality control).
- **Read–end artifacts**: artifacts from local misalignments near InDels, where the alternative allele is observed only at the beginning or end of the sequence.
- **Strand bias** artifacts: sequences supporting the variant are present only on one strand.
- **Misalignments in low-complexity regions**, such as homopolymeric regions, where errors commonly occur in sequencing by synthesis near homopolymers. After repeating the same base multiple times, sequencing platforms often substitute the first base after the homopolymer with the homopolymer base due to slippage phenomena.
- **Misalignment in paralogous regions**: misalignment in regions with paralogous sequences poorly represented in the reference genome. This type of artifact typically occurs when sequences not represented in the reference genome are aligned to the closest paralog.

### Key points summary

Variant filtering is essential to remove artifacts introduced during SGS, which can lead to false-positive and false-negative variant calls. Artifacts originate from various stages of the sequencing process, such as DNA damage, PCR errors, and library preparation, and can impact variant detection. Common artifacts include low-quality nucleotides, read–end artifacts, strand bias, and misalignments in low-complexity or paralogous regions. Effective filtering methods like frequency hard filtering and VQSR are important for reducing errors, though manual inspection of clinically relevant variants through tools like IGV is recommended to catch artifacts that may escape automatic filters.

## Visual inspection of variants and/or problematic regions

Visual inspection of alignments is a common practice to evaluate a locus in detail, especially when bioinformatic analysis has not detected suspicious events or has not flagged the presence of a hypothesized variant. Tools such as the UCSC Genome Browser, the Ensembl Genome Browser, and JBrowse are commonly used for this purpose. Among these, the IGV [12] is one of the most widely used tools and is available as a desktop application, a web application, and a JavaScript implementation that can be directly integrated into web pages [31]. IGV supports the visualization of files representing fundamental steps of SGS data analysis, from BAM, CRAM, bigWig, bigBed, to VCF. In addition to traditional alignment pileup visualization, IGV allows graphical representation of RNA-seq profiles, genomic interactions from chromatin conformation analyses, and Manhattan plots. The ability of IGV to offer these visualizations makes it an essential tool in SGS workflows, particularly for verifying the quality of specific sites and assessing the presence or absence of variants. Here are some typical examples where visual inspection via IGV can be particularly useful for determining the quality of a specific locus.

### Practical cases

**Fig. 1:**
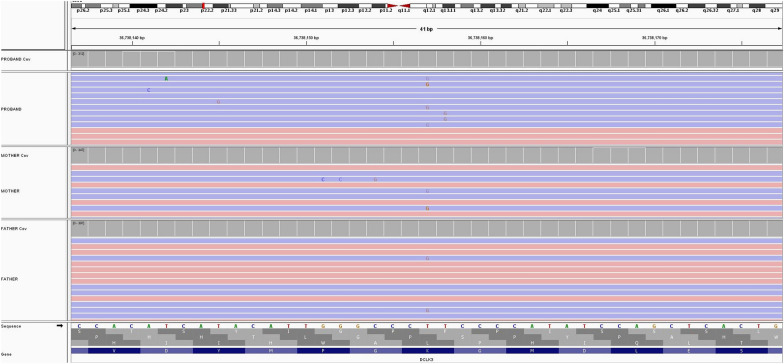
Reads alignment within a coding region of the *DCLK3* gene showing a putative T > G variant poorly supported by the reads alignment

In Fig. 1, the alignments of a trio (PROBAND, MOTHER, FATHER) are loaded into a desktop instance of IGV and viewed relative to the exonic sequence (RefSeq) of the *DCLK3* gene. For each sample, the reads are stacked below, highlighting their orientation (5'-3' red, 3'-5' blue), while the coverage profile is shown above base-by-base. Bases differing from the reference sequence are highlighted according to the changing base. The dashed vertical lines indicate a potential variant (T > G), but in several reads, G is shaded, indicating poor base quality (e.g., Phred-scaled quality score <  = 10) and consequently reduced confidence in the variant's actual presence. The chromosome where the region of interest is located is represented at the top of the figure with a red box.

**Fig. 2:**
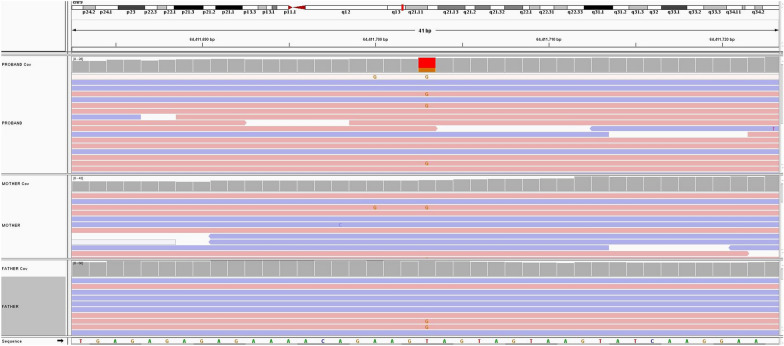
Reads alignment of an SGS experiment showing a potential T > G variant with strand bias calling

In Fig. 2, the characteristic making the variant dubious is not base quality but strand bias on 5'-3' reads, clearly shown by IGV's visualization.

**Fig. 3:**
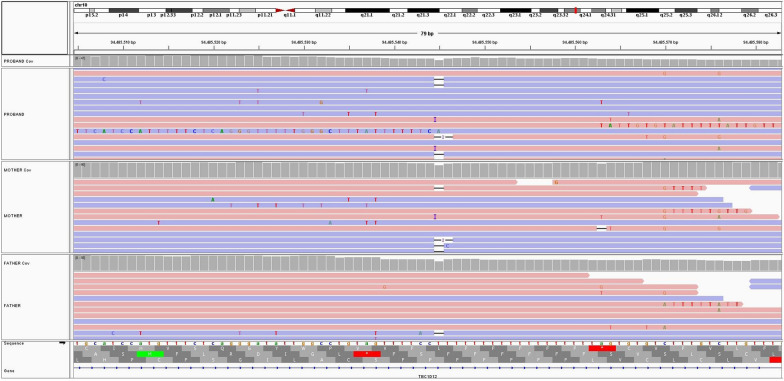
Alignment of an SGS experiment in a trio within an intronic region flanking an exon of the *TBC1D12* gene highlights misalignment issues

Figure 3 shows the recurrence of an insertion (black horizontal bar) and a deletion (blue vertical bar) artifact at the same site on different reads of the three samples. This is attributed to misalignment due to the homopolymer sequence (polyT) immediately downstream of the signal.

**Fig. 4:**
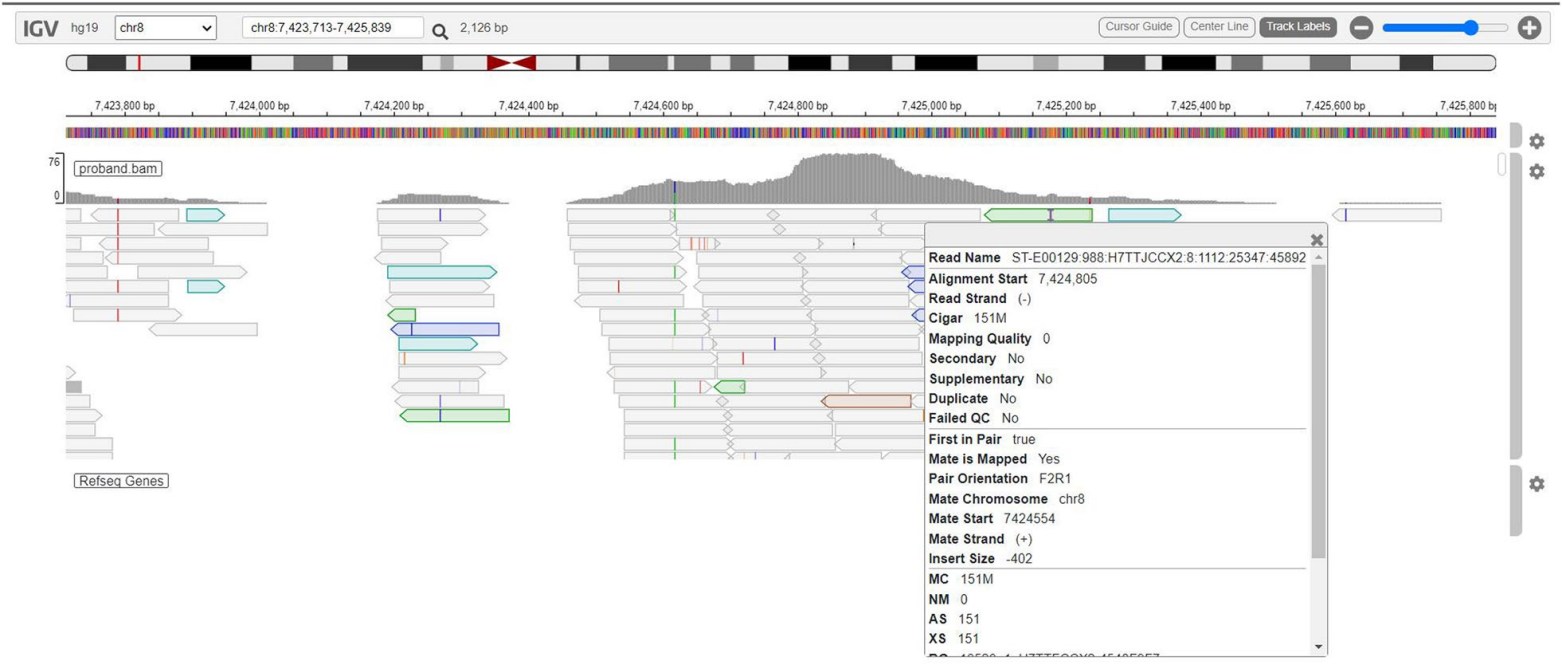
Alignment of an SGS experiment in a single sample around a low-mappability region

In Fig. 4, the IGV web app visualization (https://igv.org/app/) shows a low-mappability region represented by transparently colored reads. The BAM file parameters highlight an alignment quality of 0, indicating that these regions often harbor false-positive and false-negative variant calls owing to the high alignment ambiguity characterizing them.

### Key points summary

Visual inspection of variant alignments is a critical step in SGS workflows to assess variant quality, especially in cases where bioinformatic tools may miss or flag suspicious variants. Tools like IGV, UCSC Genome Browser, and Ensembl Genome Browser are commonly used for this purpose, with IGV being a versatile tool that supports various file formats and visualizations. Visual inspection is particularly useful for detecting issues such as poor base quality, strand bias, misalignments in homopolymer regions, and low-mappability regions. This manual review helps ensure the accuracy of variant calls and complements automated filtering processes.

## CNV/SV variant calling

This section focuses on the analysis processes for calling SVs, a category of genetic variant that poses a significant challenge in SGS. SVs include large insertions, deletions, translocations, inversions, and genomic duplications, often exceeding 1 kb in length. Unlike SNVs and small InDels, SVs can have a dramatic effect on genomic architecture and gene expression. However, their detection is complicated by their size and structural complexity. Additionally, SVs can occur in repeated or low-complexity genomic regions, making their correct alignment and interpretation difficult.

### Analysis stages

Similar to SNV and InDel identification analyses, the input for variant calling software is typically represented by BAM files derived from the alignment of sequences in FASTQ files. This process can include additional steps, such as removing PCR duplicates and recalibrating base quality scores. The standard output of these variant calling programs is the VCF format, providing details on identified genetic variants relative to the reference genome, including variant positions, types of genetic alterations, and other relevant information for research and clinical applications.

**Fig. 5:**
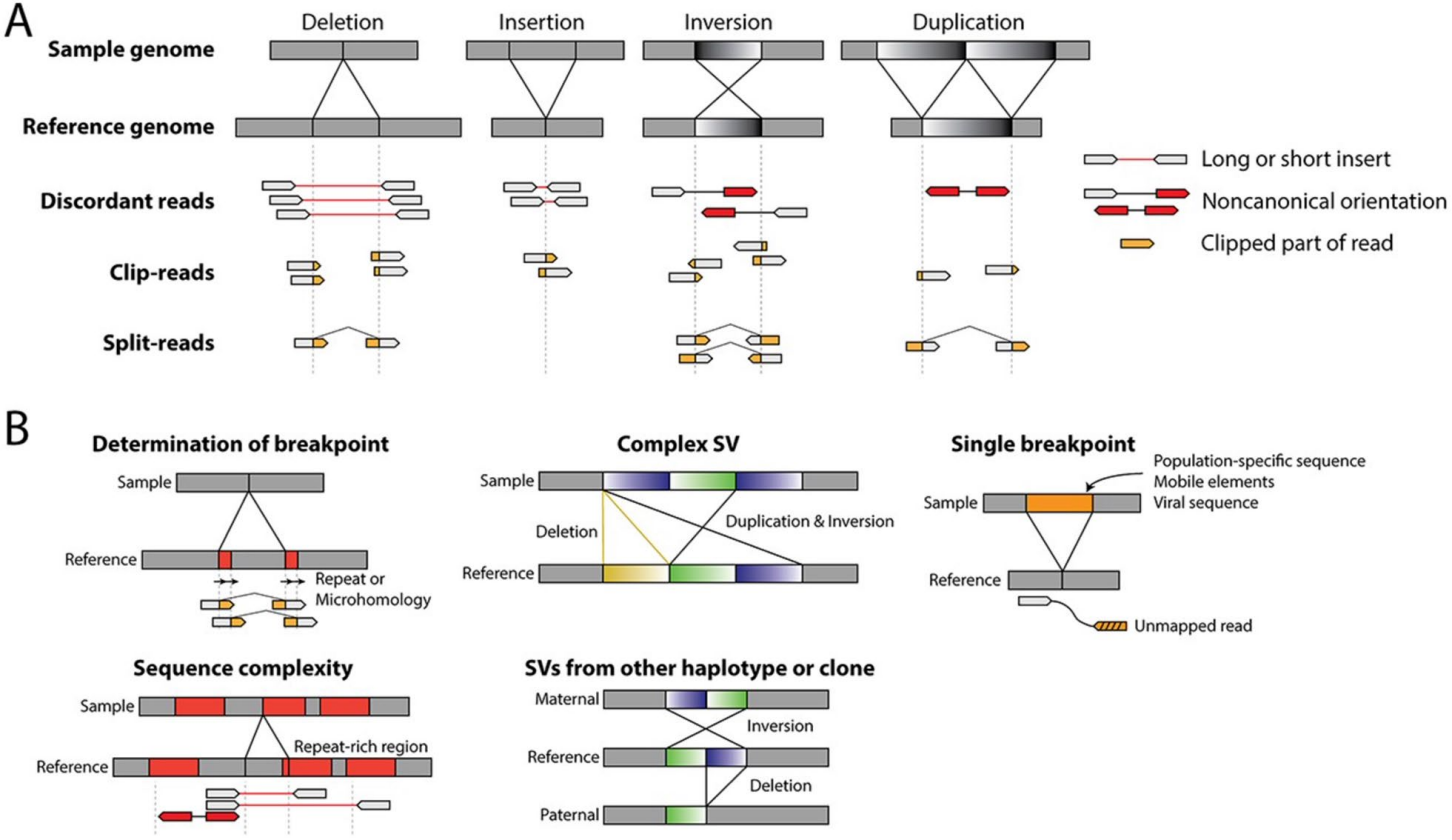
Anomalies in mapped reads and complications affect the detection of SVs. **A** Anomalies in mapped reads for different types of SVs. Sequencing reads are represented as arrows, with paired reads connected by lines. For discordant reads, a short or long insert is indicated by a red line, and an unexpected orientation of reads is indicated by red arrows. For split/clip reads, the clipped portion of the read is marked in orange. Split-read refers to a single read mapped to two distinct regions, and corresponding clipped reads are also marked in orange. For simplicity, only one forward mapped read is shown for split/clip-reads. **B** Complications in SV detection. Repetitive sequences are indicated as red boxes, whereas inserted sequences absent from the reference genome are indicated as orange boxes. These could come from population-specific sequences, mobile elements, or viral sequences Adapted from Yi et al. [32]

Following the preparatory stage of aligning sequences to the reference genome, algorithms for variant calling are used to identify specific SV types. SVs can be divided into two categories depending on whether the modification is balanced (no gross DNA loss) or unbalanced (DNA loss or gain), and their identification is based on different strategies capable of evaluating anomalies in the alignment of genomic sequences (Fig. 5).

Deletions and duplications create what are known as CNVs, which are unbalanced, whereas translocations and inversions that preserve genetic content generate balanced chromosomal rearrangements (BCRs). Historically, CNVs have been described as variations in genetic content larger than 1000 base pairs (bps). However, with technological advances improving the resolution of techniques used to identify these variants, it has emerged that individuals can present variations in genetic content ranging from 1 bp to several megabases (Mb). Owing to the rapid reduction in costs, WGS has become a feasible and sensitive method for detecting all types of SVs, including CNVs and BCRs, offering single-base resolution. This approach theoretically places no limits on the size of the SVs/CNVs that can be identified.

### Tools for CNV/SV variant calling

In recent years, there has been a surge in the development of software tools for identifying SVs and CNVs from WES and WGS [33, 34]. These tools exploit four different WGS metrics, namely, read depth (RD), split/clip read (SR), read pair (RP), and assembly based (AB) methods, each of which relies on distinct information from sequence data [35].

- **RD-based methods:** These methods are based on the principle that the coverage depth of a genomic region reflects the relative copy number of loci. An increase in copy number results in higher than average coverage, whereas a copy number loss results in lower-than-average coverage of the region.
- **SR-based approaches:** These rely on paired-end sequencing, where only one read of each pair aligns to the reference genome, while the other is unmapped or partially mapped.
- **RP methods:** These exploit discordant read pairs (DPs), where the mapped distance between read pairs significantly deviates from the average fragment size of the library, or if one or both members of the pair are aligned in an unexpected orientation.
- **AB methods:** Unlike previous approaches that rely on initial alignment to a reference sequence, these methods de novo assemble reads into contigs, which are then aligned and compared to a reference genome.

By leveraging different types of information, each of these methods presents different strengths and weaknesses: for example, RD-based methods can identify only SVs where there is a gross change in genetic content (CNVs, not BCRs). The performance of RP methods critically depends on the alignment algorithm's choice, which can be problematic for low-complexity genomic regions owing to ambiguities in correctly positioning the reads but generally can identify both CNVs and BCRs. SR methods require reads spanning the SV breakpoint, ensuring single-nucleotide resolution, but their effectiveness is proportional to the length of individual reads produced during sequencing. Finally, AB sequence analysis methods can have very long execution times and require high-performance computing resources, although they have proven more precise in identifying complex SVs. Some software uses a combination of all previous methods for more precise SV identification. A list of the most popular software in the human genomic field is provided in Table 2, while a schematic of identifying different structural variants in WES/WGS experiments and their complications is illustrated in Fig. 5.

**Table 2:** Main bioinformatics tools for identifying SVs freely available to the scientific community, sorted by year of publication, with an indication of the algorithm used: read-depth (RD), read-pair (RP), split-read (SR), discordant pair (DP), or combination of these

Tool	Version	Algorithm	Anno	Input	Output	Link
GASV	1.4	RP	2009	BAM	Custom	https://code.google.com/archive/p/gasv/source/default/source
Pindel	0.2. 5b9	RP + SR	2009	BAM	Custom	http://gmt.genome.wustl.edu/packages/pindel/
RDXplorer	3.2	RD	2009	BAM	Custom	http://RDXplorer.sourceforge.net/
CLEVER	2.4	RP	2011	BAM	Custom	https://bitbucket.org/tobiasmarschall/CLEVER-toolkit/wiki/Home
DELLY	0.8.2	RP + SR	2012	BAM	Custom	https://github.com/DELLYtools/DELLY
BreakDancer	1.3.6	RP	2012	BAM	Custom	https://github.com/genome/BreakDancer
indelMINER	N/A	RP + SR	2014	BAM	VCF	https://github.com/aakrosh/indelMINER
GRIDSS	2.5.1	RP + SR	2015	BAM	VCF	https://github.com/PapenfussLab/GRIDSS
MiStrVar	N/A	N/A	2015	Fastq/BAM	VCF	https://bitbucket.org/compbio/MiStrVar
LUMPY	0.2.4	RP, SR, RD	2016	BAM	VCF	https://github.com/brentp/smoove
PopDel	1.1.3	RP	2017	BAM	VCF	https://github.com/kehrlab/PopDel
CREST	1.0	SR	2017	BAM	Custom	https://www.stjude.org/research/labs/zhang-lab/crest.html
Manta	1.6.0	SR	2017	BAM	VCF	https://github.com/Illumina/manta
Genome STRiP	2.0	RP + SR + RD	2017	BAM	VCF	http://software.broadinstitute.org/software/genomestrip/
Octopus	0.7.4	SR	2018	BAM	VCF	https://luntergroup.github.io/octopus/
Deep Variant	1.2.0	N/A	2018	BAM	VCF	https://github.com/google/deepvariant
Tardis	1.04	RP + RD + SR	2019	BAM	VCF	https://github.com/BilkentCompGen/tardis
GROM	1.0.3	RD	2021	BAM	VCF	https://osf.io/6rtws/

The performance of these structural variant calling methods is influenced by several factors, mainly the size of the CNV/SV and the WES/WGS data coverage. For example, BreakDancer[36] can detect only deletions larger than 100 bp. Some tools have achieved excellent sensitivity at the expense of very low precision; for example, Pindel[37] exhibits the highest sensitivity among all tools but has a precision rate below 0.1%. Conversely, other tools, such as PopDel[38], adopt a more conservative approach to SV detection, achieving higher precision but with lower sensitivity for smaller deletion events. Some tools strike a good balance between precision and sensitivity, such as Manta [39], CLEVER [40], LUMPY [41], BreakDancer [36], and DELLY [42], all of which have precision and sensitivity rates above 40%. Additionally, there are substantial differences in computational resource requirements and analysis execution times among these tools, with variations of 2–3 orders of magnitude in time and necessary RAM. The primary factors influencing tool performance are sequencing depth and variant size rather than detection algorithm type (SR, RD, or RP).

In conclusion, different CNV/SV variant calling tools, each of which is based on distinct strategies, present specific strengths and weaknesses. To leverage these diverse capabilities, combining results from multiple SV identification tools into an ensemble method (also known as "ensemble learning") has been proposed. This approach has the potential to outperform individual variant calling algorithms. Several ensemble approaches, such as Parliament2[43] and FusorSV [44], have recently been proposed in the literature, demonstrating improved sensitivity by integrating the intersection or union of calls produced by different algorithms. However, establishing universal thresholds and rules for integrating these structural variant sets while maximizing both precision and variant identification remains a complex and challenging task.

For laboratories looking to integrate SV detection into routine diagnostics, we recommend referring to the recent guidelines published by the American College of Medical Genetics and Genomics (ACMG) [45]. These guidelines provide comprehensive recommendations for incorporating SV analysis via SGS, with an emphasis on validation, appropriate assay selection, and thorough reporting to ensure reliable clinical results. The ACMG's guidelines serve as a valuable resource for ensuring that the full spectrum of structural variations is accurately detected and reported in clinical settings.

### Key points summary

SVs, including large insertions, deletions, translocations, and inversions, present significant challenges in SGS due to their size and complexity. SV analysis involves aligning sequencing data to a reference genome and using various algorithms to identify different types of SVs. Common approaches to SV detection include: (1) evaluation of paired-end reads orientation and abnormal insert size (RP), (2) the presence of split and soft-clipped reads at the breakpoints of SVs (SR), (3) abnormal read depths in CNVs (RD), or (4) de novo assembly of reads before alignment (AB).

Several tools, such as GROM, Manta, and DELLY, have been developed to detect SVs, with different degrees of precision, sensitivity, and computational requirements. Combining multiple variant calling tools using ensemble approaches like Parliament2 can improve accuracy, though establishing optimal methods for integrating results remains a challenge.

## Annotation of SNV/InDel/CNV variants

The objective of the variant annotation process is to provide information for their functional interpretation. This is a crucial step, as subsequent filtering and prioritization stages are based on this information. The annotations discussed in this section pertain to constitutional/germline genomic variants in the context of monogenic or oligogenic diseases, deferring to other sources the discussion on somatic variant annotations for characterizing neoplastic lesions [46].

### Analysis stages

The variant annotation procedure involves comparing the genomic coordinates of a variant with existing or specifically created annotation databases. This process can be based solely on the position of the variant relative to the reference genome or on the sequence variation it causes. Annotation provides details on both the variant and the involved gene. At the variant level, annotation allows for obtaining and evaluating information regarding the following:

1. Variant frequency in healthy and affected individuals.
2. The predicted effect of the variant on the protein sequence or other functional regions.
3. The variant's segregation among family members, if applicable.

In general, a critical aspect of the entire annotation process is the unambiguous identification of each variant. To this end, the Human Genome Variation Society has developed an internationally recognized standard for describing variants at the DNA, RNA, and protein sequence levels, known as the HGVS nomenclature (https://hgvs-nomenclature.org/stable/). This standard is used to report variants in clinical reports, facilitating comparisons with databases and other laboratories. For SNVs, this indication is usually unambiguous. However, for multinucleotide variants, insertions or deletions, and regions with multiple isoforms or intron‒exon junctions, specific rules exist to minimize errors in variant identification and comparison.

#### Variant-level annotation

##### Presence of the variant in affected and unaffected individuals

The purpose of this first set of annotations is to check whether the variant has previously been reported in populations or has been associated with a specific phenotype.

- **Control databases**: Data on apparently healthy individuals (controls) are currently collected from a large online database called gnomAD, which aggregates sequencing results from hundreds of thousands of individuals not affected by severe pediatric conditions, including their age and sex at the time of the study. In addition to international databases, another important source of control subjects is internal laboratory databases, which, although smaller in size, have the advantage of more closely representing the genetic background of the region where the laboratory operates. The purpose of these annotations is to exclude the pathogenicity of a variant on the basis of its presence in control subjects, considering disease penetrance, transmission model, and variant frequency in controls.
- **Patient databases**: Genotype–phenotype association databases can either be genome-wide, covering the entire genome, or gene-specific, and they may be public or proprietary. Public databases like ClinVar [47] (https://www.ncbi.nlm.nih.gov/clinvar/) and OMIM [48] (http://omim.org), collect and share variant data for public use, whereas proprietary databases, such as the Human Gene Mutation Database (HGMD https://www.hgmd.cf.ac.uk/ac/index.php) [49], which compiles known gene mutations associated with human disease, require paid access. ClinVar includes annotations on individual variants, collaboratively curated by researchers and laboratories worldwide, while OMIM and HGMD primarily compile variant interpretations from the literature. In addition to these genome-wide sources, there are gene-specific databases like the Leiden Open Variation Database (LOVD https://www.lovd.nl/) [50], the Clinical and Functional Translation of CFTR (CFTR2; http://cftr2.org) for CFTR gene variants and the ENIGMA consortium for BRCA1 and BRCA2 genes (http://enigmaconsortium.org/) [51]. Most bioinformatic annotation tools automatically query genome-wide databases, although challenges may arise due to discrepancies in variant nomenclature [52].

##### Predicted functional impact

- **Effect on the protein sequence**: Transcript-level variant annotation allows estimation of the impact of a given variant on mRNA and consequently on the protein sequence. However, the same variant can involve different isoforms of the same gene (or even different genes), resulting in different functional effects on each isoform. To facilitate annotation, tools usually report only the most severe predicted effect among all possible isoforms (truncating variant > missense variant > regulatory variant https://www.ensembl.org/info/genome/variation/prediction/predicted_data.html#consequences). Another ambiguity arises from the chosen transcript model (NCBI RefSeq or Ensembl) [53]. To limit ambiguity, the MANE (Matched Annotation from NCBI and EMBL-EBI) dataset [54] was recently defined. This dataset provides a consensus transcriptome by associating a single transcript and protein sequence for each gene, prioritizing the most medically and biologically relevant transcripts.
- **Splicing alteration**: Splicing is an inherently complex process regulated by competitive interactions between splicing acceptor and donor sites and is further modulated by intronic or exonic regulatory elements. Genetic variants can affect any of these elements. Several algorithms have been developed to predict the impact of variants on splicing via sequence information. In general, the ability to predict splicing effects is greater for variants involving canonical splicing sites and significantly lower for other variants. To increase the predictive accuracy, predictions from different algorithms can be combined. For example, the dbscSNV database provides predictive scores for all possible SNVs located in consensus splicing regions (− 3 to + 8 from the 5’ splice site and − 12 to + 2 from the 3’ site) by integrating predictions from eight different tools [55]. The dbNSFP database offers predictive scores for all possible synonymous variants in the genome by integrating predictions from 43 different algorithms [56].
- **Computational models for variant impact prediction**: Numerous methods have been developed to predict the functional impact of variants, aiming to estimate whether a given variant can alter protein function or affect other functional aspects of the genome (as for regulatory or structural variants). In general, these methods integrate different information, such as phylogenetic data, amino acid biochemical characteristics, protein folding, and the involvement of functional genomic elements. Some explicitly refer to the impact of a missense variant on protein function (e.g., Polyphen and SIFT) [57, 58], whereas others more generally assess the total biological impact on the organism (CADD) [59]. In practice, protein-related annotations allow the evaluation of the impact of missense variants on coding genes, whereas other annotations can be applied to any locus. Annotation tools often report the fraction of tools that deem the variant functionally relevant, generating a prediction score. Importantly, there is some overlap between individual methods (e.g., more recent methods often integrate previous methods); therefore, these annotations should not necessarily be considered independent predictions. Additionally, these annotations do not directly indicate pathogenicity and are not specific to certain phenotypes. Recent advancements, particularly AI-assisted tools such as AlphaMissense [60]and AlphaFold[61], have revolutionized the prediction of variant impact by integrating protein structure predictions into the analysis. AlphaMissense pathogenicity scores have been made available as a public resource, and can be thus incorporated in variant annotation pipelines. These tools offer significant potential for clinical applications by providing high-accuracy predictions of the effects of missense variants on protein folding and function. However, despite their strengths, including improved accuracy in assessing protein structure, limitations remain. A recent study showed that AlphaMissense maintained consistent performance across different protein types, with lower performance mostly due to sparse or to low quality training data [62], which highlights the need for cautious interpretation in clinical settings.

**Mutational hotspot regions**: The functional role of variants may depend on the protein region where they occur, as it is known that in some genes, pathogenic variants predominantly or exclusively involve specific functional domains. Therefore, a variant within these regions is more likely to be pathogenic. In practice, hotspot regions are subgenic regions with a greater enrichment of pathogenic variants than benign ones [63, 64].

##### Experimentally-validated functional impact

In recent years, high-throughput experimental techniques have been developed to study the functional consequences of large numbers of genetic variants in parallel. These techniques, collectively known as Multiplexed Assays of Variant Effects (MAVEs), allow researchers to assess the impact of thousands of variants on specific genes or regulatory regions simultaneously. Typically, MAVEs involve generating a comprehensive library of variants for the target region (for example, through saturation mutagenesis), introducing each variant into a model system, and quantifying its effects on a specific molecular function.

Currently, MAVEs data are stored in two main databases: MaveDB [65], which covers various functional regions, including coding regions and regulatory elements such as promoters and enhancers, and SpliceVarDB [66], which focuses on assessing the impact of variants on splicing, including canonical splicing sites and deep-intronic variants. Although these resources do not yet cover all functional regions of the genome, by 2018, the number of variants validated by MAVEs was predicted to surpass the missense variants classified in ClinVar. As MAVE datasets continue to expand, they will also serve as valuable training sources for AI-based models, further enhancing in-silico predictions of variant effects.

#### Gene-level annotation

For some variants, there is no known association with a specific phenotype. In this case, information already observed for known variants in the same gene can be considered. The main annotation is the association of the gene with a monogenic disease and the described type of Mendelian inheritance. The primary databases used to derive this information are OMIM [https://omim.org/] and Orphanet [https://www.orpha.net/], which are manually curated and contain evidence from the literature and reports from condition experts. They report the associations among phenotypes, genes, and transmission patterns. Another source of gene‒phenotype associations is genetic panels, such as those in PanelApp [https://panelapp.genomicsengland.co.uk/] [67]. These panels are lists of genes associated with groups of clinical conditions (e.g., collagenopathies, retinopathies) and allow for the association of a gene with a clinical condition beyond individual diseases. Another gene‒phenotypic database is ClinGen [Welcome to ClinGen (clinicalgenome.org)] [68], which is also manually annotated. ClinGen also reports expert evaluations of the pathogenicity of specific gene alterations (such as haploinsufficiency or triplosufficiency), which is particularly relevant for annotating deletions or duplications involving entire genes. In diagnostics, an important piece of information is whether a gene has been defined as actionable, meaning it causes phenotypes that can be managed with preventive or therapeutic procedures. The most commonly used list of actionable genes is released by the ACMG [https://www.ncbi.nlm.nih.gov/clinvar/docs/acmg/] [69]. Other annotations involve the biological or phylogenetic characteristics of the gene, which can be used to implicate new genes in pathological phenotypes. These include transcriptomic or proteomic expression atlases [70, 71], gene association studies [72], and phenotypes associated with orthologous genes in model organisms [https://www.informatics.jax.org/].

### Tools for variant annotation

Several open-source tools are available for variant annotation. The most popular and widely used methods are the Variant Effect Predictor (VEP) [73], ANNOVAR [74] and SnpEff [75], whose characteristics are briefly described in Table 3**.** The nonexhaustive list includes VAT [76], VarGenius [77], AnnTools [78], Sequence Variant Analyzer (SVA) [79], VarAFT [80], Sequence Variants Identification and Annotation (SeqVItA) [81], WGSA [82], VannoPortal [83], CruXome [84], ClassifyCNV [85], CAVA [86], FAVOR [87], VarNote [88], ShAn [89]. These tools require a list of variants as input, which are usually encoded in a VCF file, and return information retrieved from various resources and databases for each genetic variant present in the file (Table 3).

**Table 3:** Main open-source bioinformatics tools for variant annotation

Tool	Version	Year	Input	Output	Link
VEP	111	2016	whitespace-separated file; vcf; HGVS identifier; Variant identifiers; Genomic SPDI notation; REST-style regions	tsv, vcf, json	https://www.ensembl.org/info/docs/tools/vep/index.html
ANNOVAR	2023 Nov 18	2010	vcf, tsv, ANNOVAR, gff3, masterVar	csv, txt	https://ANNOVAR.openbioinformatics.org/en/latest/
SnpEff	5.2 (2023–09-29)	2012	vcf, bed	vcf, bed	https://pcingola.github.io/SnpEff/

Currently, there is no precise indication regarding which annotation tools and resources to prefer; therefore, the choice is left to the laboratory. In guiding this choice, it is necessary to consider some characteristics of the software in relation to the skills and resources available. Among the main aspects to consider are as follows:

- The type of variants to annotate: most tools allow annotation of SNVs and InDels, whereas fewer software allows annotation of structural variants.
- The type of output file: many tools return a VCF file containing annotations in the INFO field. Some tools (e.g., VEP) can return annotations in tabular format, which is easier to process.
- The flexibility of annotation resources: some software allows the download of widely used resources (e.g., population frequencies from GnomAD and 1000 Genomes) during installation. Tools such as VEP also provide the ability to use and customize annotation resources according to the user’s needs, using custom files in standard formats (e.g., GFF3, bed). Additionally, some tools allow the integration of external software functions through plugins, using their output as an additional resource for annotation.
- The user interface: most tools have a command-line interface (CLI), which allows direct and flexible control of program execution. Since not all users are familiar with this type of interface, some software offers graphical user interfaces (GUIs) that simplify their use.
- Location of resources: some tools, such as VEP and ANNOVAR, can be used both locally and as web tools, i.e., accessing computational resources on a server. Similarly, the information necessary for variant annotation can be retrieved on the fly by connecting to databases or can be retrieved from previously installed local files. The use of remote resources avoids the local installation of software and annotation resources, which, depending on the databases used, can require significant storage space. However, this execution mode is usually slower, and the number of variants that can be analyzed may be subject to limitations.

Comparative studies have been conducted to establish the performance of annotation tools and to highlight potential issues. These studies typically refer to sets of variants of clinical interest whose annotation is manually reviewed by a panel of experts.

Different annotation tools may attribute different functional impacts to the same variant.

A study aimed at comparing the performance of VEP and ANNOVAR using the same transcriptome model[53] revealed that the two tools assign the same functional impact to 65% of genomic variants and to 87.3% of variants located in exons. A greater degree of discordance between the two tools is thus detected for variants located in splicing sites, intergenic regions, intronic regions, and sites coding for noncoding RNA. In analyzing the discrepancies between the two tools, the authors identify an effect of the prioritization algorithms (especially for frameshift and stop gain/loss variants) and annotation algorithms (for splicing variants) used by the two software programs. In most cases, where the two tools are discordant, manual verification indicates greater accuracy of VEP in annotating the functional impact.

With respect to the HGVS nomenclature, VEP and SNPEff appear to have comparable efficiencies [52], whereas ANNOVAR was found to be less accurate than VEP in an independent study [90].

In general, the tools are more accurate in the nomenclature of SNVs than in the nomenclature of insertions and deletions, especially when the variant is indicated at the transcript level. To overcome these ambiguities, it is preferable to always indicate variants at the genome level.

For those looking to deepen their understanding of the interpretation and classification of sequence variants in clinical settings, we recommend referring to the guidelines developed by the ACMG [91]. These guidelines, formulated in collaboration with the Association for Molecular Pathology (AMP) and the College of American Pathologists (CAP), provide a comprehensive framework for interpreting sequence variants, categorizing them as 'pathogenic', 'likely pathogenic', 'uncertain significance’, 'likely benign', or 'benign'.

### Key points summary

The goal of variant annotation is to provide functional information on genetic variants, critical for interpreting and prioritizing variants in clinical and research contexts. The process involves comparing genomic variants to annotation databases, identifying their presence in healthy and affected populations, predicting their functional impact, and assessing potential effects on protein sequences or splicing. Tools like VEP, ANNOVAR, and SnpEff are commonly used for annotation, each offering different capabilities, such as handling variant types, providing flexible resources, and varying user interfaces. The accuracy of annotation tools can differ, particularly for complex variants like insertions or deletions. Manual verification often indicates higher accuracy for VEP in functional impact prediction.

## Analysis of sequencing data derived from mitochondrial DNA

Traditional bioinformatic analysis pipelines for SGS data obtained from WES and WGS allow the identification of various types of genetic alterations. Unfortunately, most tools available for the analysis and annotation of genetic variants are not optimized to include variants present in mtDNA in the output files. This is due to a peculiar characteristic of the mtDNA called heteroplasmy, i.e. the presence of more than one type of mitochondrial genome within a cell. In fact, unlike the nuclear genome, which is only present in two copies, there can be ∼1,000 to 10,000 copies of mtDNA in most somatic cells. Thus, the mtDNA can exist in a state of heteroplasmy, where there is variation in the sequence of the different mtDNA molecules within a cell, or homoplasmy, where all mtDNA share the same sequence. The proportion of mutant and wild-type molecules is often referred to as the heteroplasmy percentage or heteroplasmy frequency.

Despite this, it is possible to extract this type of information from raw SGS sequencing data (WES or WGS) via dedicated bioinformatic tools that can be operated with standard hardware and expertise already available in genetics laboratories conducting SGS.

Starting from the FASTQ files of samples sequenced via WES and WGS, it is possible to perform variant calling on the mitochondrial chromosome (chrM), including both homoplasmic and heteroplasmic variants (even at low percentages > 2–3%).

### Analysis stages

The alignment phases used to generate BAM files are the same as those used in a classic analysis pipeline for WES or WGS. The only difference is in aligning the reads contained in the FASTQ files to the human mitochondrial genome (revised Cambridge Reference Sequence—rCRS, NCBI NC_012920.1) instead of the complete human genome, which uses the same commands. From the generated BAM files, it is then possible to call mitochondrial variants through bioinformatic tools that can be used locally with command strings, as described in the following paragraph.

### Tools for identifying mitochondrial DNA variants

Unlike the nuclear genome, where variants are typically present at expected Variant Allele Frequencies (VAFs) of approximately 50% (heterozygous) or 100% (homozygous), mtDNA heteroplasmy poses a unique challenge. Heteroplasmic variants can exist at any allele fraction because each cell contains numerous mitochondrial genomes, which may differ from one another. Standard bioinformatic variant callers often discard low VAFs, assuming them to be sequencing artifacts. However, in mtDNA analysis, even low VAFs are significant and must be accurately identified. Specialized tools, often adapted from those used in somatic cancer mutation detection, are required to call mtDNA variants at all VAF levels to ensure comprehensive variant detection.

Only in recent years, specific programs have been developed for calling mitochondrial variants, each with slightly different characteristics, and optimized to identify types of variants peculiar to the mitochondrial genome (Table 4).

- **Mutect2:** A widely used tool particularly suitable for calling heteroplasmic SNV and InDel variants, initially designed for somatic variants (tumors).
- **Mity:** This method performs calling of heteroplasmic SNVs and InDels, is very sensitive for WGS data, performs extensive variant annotation, and has been validated in clinical studies. The required input is an aligned data file (BAM) from which homopolymeric regions (m.302–319 and m.3105–3109) are filtered [92].
- **MToolBox:** This tool performs calling of heteroplasmic SNVs and InDels and proceeds to their annotation. This method is suitable for use with WGS and WES data, including off-target reads from WES. The input can be either aligned BAM files or unaligned FASTQ files [93].
- **Mt-DNA server:** A cloud-based application as part of the mitoverse suite, with an intuitive interface for heteroplasmic SNV and InDel calls. It recognizes and flags low-complexity regions and known nuclear mitochondrial DNA segments (NUMTs). It accepts aligned or unaligned WES, WGS, or mtDNA-only data as input [94].
- **MitoScape:** A pipeline for calling heteroplasmic SNVs and InDels from WGS data, primarily designed for complex diseases. It uses a new machine learning approach for extremely accurate calling and removal of so-called NUMTs. The performance is better than that of the MToolBox and Mt-DNA servers, and it can be used to estimate mtDNA copy number.
- **MitoHPC:** A pipeline for measuring mtDNA copy number (as a ratio of mtDNA coverage) in WGS data. It also calls and annotates heteroplasmic SNVs and InDels, performs additional circularized alignment, and generates an individual-specific mtDNA "reference" sequence, reducing the identification of false positive variants. Additionally, it flags homopolymeric, hypervariable regions, and NUMTs [95].
- **eKLIPse:** Designed to identify multiple breakpoints of multiple deletions and generate Circos plots [96].
- **MitoSAlt:** This allows the quantification of deletions and duplications on the basis of the analysis of sequences with breaks. It includes a second alignment phase to identify broken sequences in mapped and unmapped data [97]. It is particularly suitable for WGS data.

**Table 4:** Tools for mitochondrial DNA analysis

Tool	Version	Year	Input	Output	Link
Mutect2 (mitochondria-mode)	4.5.0	2013	BAM	VCF	https://gatk.broadinstitute.org/hc/en-us/articles/360042477952-Mutect2
Mity	1.0.0	2022	BAM	VCF	https://github.com/KCCG/mity
MToolBox	1.2.1	2014	BAM, Fastq	VCF	https://github.com/mitoNGS/MToolBox
Mt-DNA Server (mitoverse)	2.0.1	2016	BAM	VCF,.csv	https://mitoverse.i-med.ac.at/index.html#!
MitoScape	1.0	2021	BAM	VCF	https://github.com/larryns/MitoScape
MitoHPC	9	2022	BAM	.tab	https://github.com/dpuiu/MitoHPC
eKLIPse	2.1	2019	BAM	.csv,.png	https://github.com/dooguypapua/eKLIPse
MitoSAlt	1.1.1	2020	Fastq	.bed,.tsv,.pdf	https://sourceforge.net/projects/mitosalt/

For those who wish to delve deeper into the role of mtDNA variants in human diseases and strategies for their analysis from SGS data, we recommend reading the following publications: Stenton & Prokisch [98] and Schon et al. [99]

### Key points summary

mtDNA variants, including homoplasmic and heteroplasmic variants, can be identified from WES and WGS data using specialized bioinformatic tools. Although traditional sequencing pipelines do not typically include mtDNA in their output, variants can still be called by aligning reads to the mitochondrial genome. Tools like Mutect2, Mity, and MToolBox are optimized for detecting mtDNA-specific variants such as SNVs and InDels. Advanced tools like MitoHPC and MitoScape offer additional capabilities, such as mtDNA copy number estimation and removing false positives due to NUMTs. These tools support both clinical and research applications in analyzing mitochondrial variants linked to human diseases.

## HLA allele typing from SGS data

HLA class I and class II genes are the most polymorphic genes in the human genome. Because of this, traditional SGS variant calling methods often perform poorly at this locus located on chromosome 6. Accurate variant calling in the HLA region (HLA typing) therefore requires specifically designed algorithms. HLA typing generally focuses on six classical HLA genes (HLA-A, HLA-B, HLA-C, HLA-DRB1, HLA-DQB1, and HLA-DPB1). In addition to the classical HLA genes, there are other less studied, nonclassical HLA genes (such as HLA-E, -F, and -G). HLA genes present the highest degree of polymorphism (the largest number of registered alleles) and are the most clinically relevant. Indeed, several hundred diseases have now been reported to occur more frequently in individuals with particular HLA genotypes. These diseases comprise a broad spectrum of immune-mediated pathologies involving all major organ systems, some malignant tumors, infectious diseases, and, more recently, adverse reactions to specific drugs and tumors [100, 101].

### HLA typing methods: traditional vs. SGS

The rapid development of SGS technologies has shifted attention to HLA typing using exome or genome sequencing data (WES or WGS), rather than traditional HLA typing methods that focus exclusively on the HLA region and involve a laborious enrichment phase.

WGS and WES produce sequencing data that are not limited to one or two exons encoding the antigen recognition domain (ARD). This allows for the identification of the sequences of all exons (in both WES and WGS) and introns and untranslated regions (in WGS only), often resolving the ambiguity problem. Another significant advantage of using WES or WGS compared with data generated exclusively for HLA typing is the ability to integrate HLA typing into a broader genetic analysis. WGS/WES data are generated for multiple purposes and, in many cases, are already available, making them a more versatile and efficient approach.

### Tools for HLA typing from SGS data

One of the first algorithms developed for HLA typing from SGS data was HLAminer [102]; however, new, better-performing algorithms are continuously being published [103]. According to a comparative study [104], the tools that proved most accurate were HLA-HD [105] and OptiType [106] for class II and class I HLA genes, respectively, although tools such as T1K [107], recently published and developed by the same author of BWA, have not yet been included in these comparisons and promise potentially superior performance.

These algorithms often start with a common phase of filtering out sequences, as in a typical WES/WGS dataset, not mapping to the region of interest. This phase allows reducing the file size and speeding up the typing process.

HLA typing algorithms can be roughly divided into two groups: de novo assembly-based methods and methods that directly align to a reference genome.

- **De novo assembly-based methods:** These methods first construct a consensus sequence for HLA genes from the input reads without using a reference genome. After the sequences have been assembled into consensus sequences, they are compared with reference HLA sequences for allele assignment. The algorithms that use this approach include HLAminer [102], ATHLATES [108], HLAreporter[109] and xHLA [110]. Despite being defined as de novo assembly based algorithms, they still require an alignment/comparison phase with reference HLA sequences such as the IPD-IMGT/HLA database (https://www.ebi.ac.uk/ipd/imgt/hla/).
- **Direct alignment-based methods:** These methods use various alignment algorithms, including BWA-MEM, BOWTIE, or Novoalign, and a reference genome, often the aforementioned IPD-IMGT/HLA database. The final phase of the HLA typing process consists of determining which HLA alleles best explain the sequences organized through assembly and/or alignment.

HLA typing tools employ various references to deduce HLA alleles, but most of them use the IPD-IMGT/HLA database and attempt to identify alleles from this database that best represent the selected SGS sequences. The PHLAT [111] and Polysolver [112] tools use a Bayesian approach. ATHLATES [108] identifies alleles on the basis of their Hamming distance, whereas OptiType [106] uses an allele scoring matrix. Other tools, such as HLA*LA [113], use a graph data structure-based approach. Instead of finding the best alignment against a linear reference, it performs HLA allele inference by computing the most likely path through a graph structure.

### Optimization and accuracy in HLA typing

A study from 2017 analyzed platform-specific typing errors for a series of tools [114]. One of the results was that the OptiType frequently produced typing errors for several classical HLA alleles. To avoid this type of bias, it is possible to adopt an ensemble approach that considers the predictions of multiple tools to produce an overall final prediction with increased accuracy.

Individual alleles do not have a constant frequency but differ significantly both within a population and between ethnic groups. The Allelefrequencies.net (https://www.allelefrequencies.net/) website provides information on allele frequencies from various studies on the world population. This information can be used by typing algorithms to provide more accurate results.

### Key points summary

HLA typing is crucial for identifying variants in polymorphic HLA class I and II genes, which are associated with immune-mediated diseases, tumors, infections, and drug reactions. Traditional variant calling methods perform poorly in this highly polymorphic region, and specialized algorithms are required for accurate HLA typing. SGS-based HLA typing from WES or WGS data is advantageous, as it covers entire exons and introns, resolving ambiguities and allowing integration into broader genetic analyses. HLA typing tools use either de novo assembly or direct alignment methods, relying on reference databases like IPD-IMGT/HLA. Tools such as xHLA, HLA*LA, and HLA-HD are the most commonly used, with ensemble approaches improving accuracy.

## Identification of regions of homozygosity from SGS data

**Fig. 6:**
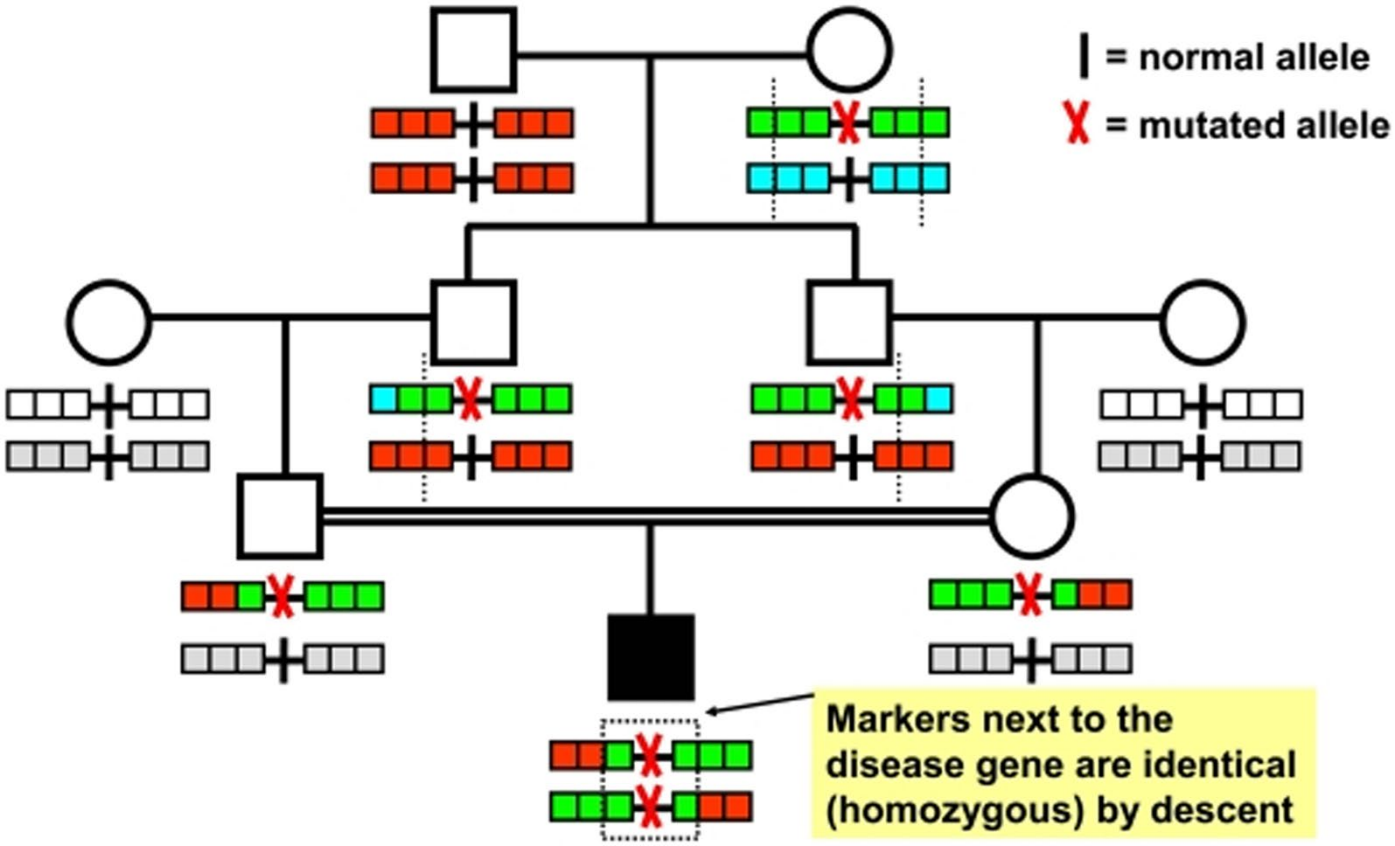
Homozygosity Mapping in recessive diseases. An individual affected by an autosomal recessive disease whose parents are consanguineous will most likely be homozygous (identical) by descent for the disease allele, as it can pass from a common ancestor through both the paternal and maternal lines, making the child homozygous for the mutation. The chromosomal segments surrounding the disease gene locus are shown with 3 marker positions on both sides. The different marker alleles are represented by different colors. Although for each parent‒child succession, there is the possibility of a crossover (dashed line) occurring in the parents' gametes, there is a high probability that in the affected child, the consecutive markers surrounding the mutation have not recombined and are identical (homozygous) by descent (from Hildebrandt et al. [117])

ROHs are defined as tracts of the genome characterized by the presence of stretches of homozygous genotypes at consecutive polymorphic DNA marker positions. Their identification has historically been linked to 'homozygosity mapping', one of the most robust methods for identifying new recessive disease genes (Fig. 6). In particular, this method is widely used in families with declared or presumed consanguinity to study autozygosity, i.e., a particular type of homozygosity that results from the cooccurrence, at a given locus, of the same allele derived from a common ancestor [115, 116].

### ROH identification methodologies: traditional vs. SGS

Traditionally, ROH identification has been based on the use of microarrays with a high density of single nucleotide polymorphisms (SNPs). SNP arrays allow simultaneous genotyping of a large number of SNPs, generally in noncoding regions, which have a high degree of heterozygosity in the general population. With the advent of SGS technologies, it has become possible to expand the map of the human genome, and today, various population databases contain information for millions of polymorphisms. For example, the 1000 Genomes Project alone has led to the genotyping of approximately 88 million SNPs, building a genetic map with an average distance between SNPs of 73 bp. However, the use of these genetic maps in the context of SGS, as an alternative to SNP arrays, was not immediate. The first SGS approach used on a large scale was targeted resequencing (pre-sequencing panels and WES), which primarily identifies exonic SNPs that can be very distant from each other. Moreover, the algorithms and related software developed for SNP arrays are not ideally suited for analyzing SGS data owing to the background noise characteristic of this sequencing method.

### Tools for ROH identification by SGS

In 2011, it was demonstrated for the first time that WES data contain a sufficient number of informative SNPs to allow reliable homozygosity mapping, and subsequent works have confirmed that ROHs can be correctly identified from these data [118–121].

Initially, software programs used for SNP array data, such as PLINK[122] and GERMLINE [123], were adapted with specific options. These programs use an algorithm called a sliding window that allows chromosomes to be scanned by moving a fixed-size window along their entire length in search of tracts of consecutive homozygous SNPs. Another program that uses this method is HomozygosityMapper [124]; this was developed for SNP arrays but has been modified to take VCF files as input and output a bed file with the genomic coordinates of the identified ROHs. Among the advantages of this program are ease of use, as it is web-based, provides intuitive graphical visualization, and uses the VCF file as input [124]. In contrast, to use PLINK and GERMLINE, it is necessary to create files in specific formats and have bioinformatics skills.

Subsequently, several ad hoc programs were developed, and the use of the B allele frequency (BAF) was introduced as a measure of genotypic state. BAF is calculated as the ratio between the number of reads carrying the B allele (alternative allele) and the total number of reads at a given polymorphic position, and its use has advantages over genotype analysis. In fact, BAF calculation does not require steps with a high computational cost, such as those necessary for variant calling, which is generally performed only for variant sites of the genome, and reference genotypes (0/0) are not reported, for which further analysis would be necessary [120].

The algorithm most commonly used by programs that employ BAF to estimate homozygosity is based on Hidden Markov models (HMM). The first tool that used this approach and was developed specifically for WES data was H3M2 [125]. In particular, this program uses a heterogeneous HMM algorithm that incorporates the distance between consecutive SNPs to probabilistically discriminate the heterozygous/homozygous state. This new approach has allowed the identification of ROHs of any size with high specificity and sensitivity, not just large ROHs (> 1.5 Mb) closely associated with consanguinity.

Several programs that use BAF and HMM models were subsequently developed (Table 5). The most commonly used methods are BCFTools/RoH [126], HOMWES [127], SavvyHomozygosity and SavvyVcfH homozygosity [128], Automap [129] and ROHMM [130]. All these programs process VCF files, whereas H3M2 and SavvyHomozygosity require BAM files; Automap is also accessible via a web interface. Given the ease of use of many of these programs, it is advisable to include ROH identification in the standard pipeline. Their analysis is important not only in the context of consanguinity, and in this case, it is no longer necessary to use two tools (SNP arrays for homozygosity and exomes to identify variants), but it is useful for estimating possible undeclared or unknown kinships and for identifying uniparental disomies.

**Table 5:** Tools for ROH calling

Tool	Version	Year	Input	Output	Link
PLINK	1.19	2007	Format PLINK	Format PLINK	https://zzz.bwh.harvard.edu/plink/
GERMLINE	15.3	2009	Format GERMLINE	Format GERMLINE	http://gusevlab.org/projects/germline/
HomozygosityMapper	na	2012	Genotype	BED file, visual inspection	https://www.homozygositymapper.org
H3M2	2017–20-10	2013	BAF – only BAM file	BED file	https://sourceforge.net/projects/h3m2/
BCFTools/RoH	1.19	2016	BAF	TXT file	https://github.com/samtools/BCFtools
SavvyHomozygosity	1	2017	BAF – only BAM file	BED file	https://github.com/rdemolgen/SavvySuite
SavvyVcfHmozygosity	1	2017	BAF	BED file	https://github.com/rdemolgen/SavvySuite
HOMWES	0.107.0	2016	BAF	BED file	https://github.com/derijkp/genomecomb
Automap	1	2021	BAF	TXT file e PDF file	https://github.com/mquinodo/AutoMap/
ROHMM	1.0.4b	2022	BAF	BED file	https://github.com/gokalpcelik/ROHMMCLI

### Key points summary

ROHs are stretches of the genome with consecutive homozygous genotypes, often used in homozygosity mapping to identify recessive disease genes. Traditionally, ROHs were identified using SNP arrays, but the rise of SGS technologies has enabled their detection from WES and WGS data. Several tools, such as PLINK, GERMLINE, and H3M2, can identify ROHs, with more recent methods incorporating BAF and HMM for improved accuracy. These tools provide insights into consanguinity, uniparental disomies, and potential kinship, making them valuable additions to standard sequencing analysis pipelines.

## Third-generation sequencing

TGS, which is based on long-read technology, has recently undergone rapid development, significantly improving in terms of DNA library preparation and sequencing quality, and the major impetus for investing resources in optimizing TGS has been overcoming the limitations inherent in SGS, which are based on short reads. In the field of medical genetics, TGS is primarily used to identify sequences characterized by SVs and sequences with expansions/contractions of repeated units (e.g., triplet expansions), which are very difficult to accurately identify with short-read SGS. Consequently, most bioinformatic tools developed for TGS data focus on detecting these types of alterations. In addition to studies related to human genetic diseases, TGS is widely used in multiple fields, such as de novo genome sequencing of animal or plant organisms and microorganisms, including bacteria and viruses.

The use of short-read approaches can highlight several critical issues in sequencing genome regions characterized by complex rearrangements, regions with high homology, and a high rate of repetitions. Despite the use of sophisticated bioinformatic algorithms, accurate mapping or assembly of sequences from regions characterized by SVs, repeated sequences, sequences with high guanine‒cytosine (GC) content, or sequences with multiple homologous elements within the genome is often impossible. These regions can be difficult to analyze owing to issues such as the lack of or altered representation of certain genomic regions during DNA library preparation or errors at the sequence alignment level, leading to subsequent errors in variant calling, particularly structural ones. Additionally, short-read SGS often results in the loss of phase information for multiple variants within the same gene. Another limitation of this approach is its dependence on a reference genome, which can be problematic when detecting SVs in complex genomic regions that are highly specific to an individual or a specific population [131].

With the recent success in identifying difficult-to-analyze DNA sequences and completing "gaps" in the human genome sequence [132], TGS has demonstrated its ability to overcome the limitations of short-read approaches, even in the study of human genetic diseases. The main advantage of TGS lies in the generation of very long sequences, with average lengths exceeding 10 kb, obtained from the reading of single native DNA molecules. These methods are based on real-time sequencing processes, where both DNA library preparation and sequencing occur without PCR-based amplification, thus avoiding errors and biases associated with this method. The absence of PCRs preserves the DNA in its native form, allowing TGS sequencers to detect base modifications, such as methylation, a possibility that is entirely precluded with short-read approaches.

WES is now typically chosen as a first-level test for many genetic diseases and has significantly advanced genetic testing and diagnostics, enabling the discovery of new disease genes at an unprecedented rate. However, for many patients who have undergone WES or even WGS, the genetic cause of their disease remains unknown. Recent TGS-based WGS studies have shown that a single individual's genome can contain more than 20,000 SVs (> 50 bp) and thousands of indels (< 50 bp), which have escaped detection by short-read analyses.

In patients affected by genetic diseases, these hidden variants could disrupt known or candidate genes or induce alterations in their expression levels. Additionally, SGS approaches based on short reads have several limitations in capturing and sequencing GC-rich areas, which typically have low coverage. It is estimated that regions characterized by low or zero coverage with high GC content exceed hundreds of megabases and include areas with high gene density, potentially hosting genetic alterations underlying various diseases.

### PacBio sequencing

The PacBio sequencing method, also known as single-molecule real-time (SMRT) sequencing, was the first nanosensor-based technology introduced by Pacific Biosciences (PacBio) in the early 2010s [133]. PacBio technology exploits the properties of DNA synthesis and allows the identification of molecules with an average length greater than 10,000 nucleotides [134]. Unlike SGS techniques, SMRT sequencing is based on the immobilization of a DNA polymerase in each well of a specially designed silicon chip (SMRTcell), while DNA is the mobile molecule [135].

Synthesis reactions are measured within thousands of wells containing microscopic sensors, called "zeromode waveguides" (ZMWs). The ZMW sensors prevent the propagation of light emitted by the incorporation of labeled dNTPs in the elongated strand, whereas a system consisting of a laser and a camera records the signal generated by the sensors. PacBio platforms allow the simultaneous detection of thousands of single-molecule sequencing reactions. For synthesis, a special circular double-stranded DNA adapter, called SMRTbell, is needed; sample preparation therefore includes connecting this molecule to the target DNA [135].

Over the years, many SMRT sequencers have been designed and marketed by PacBio; however, the first devices, such as PacBio RS II, have been progressively replaced by instruments of the Sequel System family that share optimized features, such as improvements in sequencing chemistries, automation, runtime monitoring, touchscreens, integrated software and control of the capacity of each run. The Sequel System, the first member of the family released in 2015, is capable of producing a total data output of up to 7.6 Gb [136]. The Sequel II system is capable of performing up to 30 h of sequencing and offers eight times the sequencing capacity of the previous system, with the advantages of greater accuracy and significantly reduced cost. The Sequel IIe system is PacBio's most recent platform, which performs sequencing in 8 million ZMWs and generates up to 4,000,000 sequences in a single run with a total output of up to 500 Gb.

The files used for data storage are based on the "hierarchical data format 5" (HDF5) standard. HDF5 files contain all the information generated by a sequencing run, including real-time kinetic characteristics, and therefore differ from the classic FASTQ output provided by previous generation SGS approaches; for the analysis of these data, the use of new bioinformatic tools is therefore necessary [137]. The first step for data analysis is the conversion of raw data into a nucleic acid sequence (base calling). In PacBio raw files, the translation of kinetic information into nucleotide sequences follows the "circular consensus sequencing" (CCS) workflow and produces high-precision sequences (> 99%), called HiFi [138]. Updates to the base calling software released in recent years have increased the quality of the reads produced, so it is expected that, together with the rapid improvement of technologies, further software development may still reduce the error rate associated with this technology. The quality control phase, which is based on predefined metrics, classifies the sequences into high- and low-quality reads. LongQC is a useful tool for evaluating the quality of reads from TGS data [139].

### Nanopore sequencing

In 2015, Nanopore sequencing was commercially introduced by Oxford Nanopore Technologies (ONT) through a portable MinION sequencer, which is slightly larger than a USB stick, followed by new high-yield sequencer models, called GridION and PromethION. The basic principle of Nanopore sequencing consists of passing a single strand of a DNA molecule through a Nanopore fixed on a membrane characterized by a potential difference between one side and the other. The various DNA strands are passed through the pore by a motor protein, and the conformational changes of the pore occur differently depending on the base that passes through it. The passage of the bases induces an opening of the pore, which causes a variation in the potential with consequent formation of a measurable electrical signal from sensors; this signal is subsequently converted into a DNA sequence. For Nanopore sequencing, there is no limitation regarding the read length; if not the size of the DNA molecules themselves, good-quality DNA samples will yield longer sequences, whereas degraded and/or fragmented samples will generate shorter sequences. On average, in good-quality DNA samples, the sequences generated by this type of sequencing are greater than 10 kb in length, but ultralong sequences greater than 1 Mb in length have also been reported.

Among the available sequencers, MinION is the smallest and allows the sequencing of up to 50 Gb, with greater throughput, and the GridION and PromethION sequencers are available and are capable of sequencing up to 250 Gb and 14 Tb, respectively. One of the major limitations of Nanopore sequencing lies in the low quality of the sequenced bases; the raw reads, in fact, generated with the R9 version flow cell, were characterized by an accuracy of approximately 96%, with errors represented mainly by false deletions and homopolymers [140, 141]. Recently, however, a new flow cell, version R10, was developed with chemistries capable of achieving a sequencing performance that allows an accuracy close to 99%, which is very similar to that obtained with short-read SGS methods.

### Phases and tools of alignment and variant calling

At the bioinformatics level, the main alignment tools for sequences obtained with Nanopore technology are Minimap2 [142], which was developed to align sequences containing large insertions or deletions, and ngmlr [143], which was developed to align sequences characterized by different types of SVs. Both tools generate.bam files from.fastq files.

With respect to variant calling, one of the most widely used tools is “sniffles” [143], which were developed in combination with the "ngmlr" aligner. It can also be used starting from bam files obtained with Minimap2 and allows the generation of VCF files containing various types of information, among the most important being the type of alteration (e.g., deletion or duplication), the start and end coordinates on the genome, and the number of sequences (coverage) containing such alterations. Recently, Straglr [144], a specific tool for identifying tandem repeat expansions from alignments obtained with Minimap2, was developed.

### Key points summary

TGS using long-read technology has advanced significantly, overcoming limitations of short-read SGS, particularly in identifying SVs and repetitive sequences. TGS enables sequencing of long DNA fragments (10 kb or more), providing better accuracy in complex regions like those with high GC content or SVs. PacBio and ONT are the main TGS platforms, with PacBio’s SMRT sequencing and ONT’s Nanopore sequencing offering advantages in reading long sequences, detecting base modifications, and improving accuracy with recent updates. Tools like Minimap2 and sniffles are used for alignment and variant calling, and TGS is increasingly used in genetic disease research and de novo genome sequencing.

## Techniques for identifying methylated DNA regions

DNAm, among the most studied epigenetic modifications, primarily involves the addition of a methyl group to the 5' carbon of cytosines, generally in the context of a CpG dinucleotide. Analysis of the methylation state of specific genomic regions has diagnostic value in some genetic diseases (e.g., fragile X syndrome and imprinting disorders). Furthermore, in recent years, the analysis of methylation profiles at the genomic scale has also proven to be a useful diagnostic tool. In fact, disease-specific DNAm profiles, known as DNAm signatures or episignatures, are stably reproduced in individuals affected by a significant number of neurodevelopmental disorders[145] and can support either the clinical diagnosis of patients carrying variants of uncertain significance (VUS) or uninformative molecular findings.

A recent study recommends the use of a standardized four-level interpretation scale for episignature testing: negative, inconclusive, positive with moderate confidence, and positive with high confidence [146]. High-confidence positives offer strong diagnostic evidence, while moderate-confidence results suggest the need for further testing. Inconclusive results should be interpreted with caution, prompting additional investigation, and negative results, while not ruling out pathogenicity, can still support diagnosis when combined with other clinical data. The aim of these recommendations is to standardize reporting practice and enhance the diagnostic utility of DNAm episignature testing, thereby improving clinical outcomes.

Moreover, analysis of the methylation state of imprinting control regions, conducted in parallel with the use of SGS methods, has proven useful in the diagnostic process of multilocus imprinting disturbances (MLIDs) [147].

### Experimental methods

To date, the gold standard method for analyzing the DNAm state involves treatment with sodium bisulfite. This reagent induces oxidative deamination of unmethylated deoxycytosines to deoxyuracils, leaving methylated deoxycytosine residues unchanged. The DNAm state can therefore be examined on a genomic scale through the use of methylation arrays or direct DNA sequencing.

- **Methylation arrays:** The methylation arrays currently in use allow examination of the methylation state of numerous sites (850,000 with the Illumina EPIC BeadChip; 950,000 with the Illumina EPICv2 BeadChip), which are mostly located in CpG contexts and are representative of the methylation state of known regions (CpG islands, promoters, and enhancers). To date, methylation arrays constitute an economical and scalable strategy for characterizing methylation profiles in large cohorts. Methylation arrays are commonly used in epigenome-wide association studies (EWASs) and can also analyze DNA samples extracted from formalin-fixed, paraffin-embedded (FFPE) tissues. Methylation arrays also allow the determination of the number of gene copies in the analyzed region and can, therefore, reveal CNVs [148].
- **Direct sequencing methods:** These methods allow the analysis of DNAm at the whole-genome level (WGBS, whole-genome bisulfite sequencing) or specific regions of interest appropriately enriched through the use of restriction enzymes (reduced representation bisulfite sequencing, RRBS) or capture kits (methyl capture). Despite being more costly than arrays are, sequencing methods offer higher genomic resolution, allowing determination of the methylation state of all cytosines in the regions of interest regardless of the sequence context.
- **TGS:** The introduction of TGS techniques promises to revolutionize DNA methylation analyses. This new sequencing technology allows the direct identification of modified bases, avoiding treatment with sodium bisulfite and associated DNA degradation.

### Analysis stages

The main phases of the methylation data analysis are as follows:

1. Quantification of the methylation state of individual cytosines;
2. QC;
3. Filtering and normalization;
4. Identification of differentially methylated sites and regions.

Each of these steps must be adapted to the experimental strategy used (array, SGS, or TGS) and therefore to the starting data. For arrays, the analysis starts from IDAT files, in which the intensity of the hybridization signal for each oligonucleotide probe present on the array is stored in a compressed manner. For arrays, the methylation state of individual sites is quantified as a beta value, i.e., as the ratio between the intensity recorded for probes complementary to the cytosine in the methylated state (M) and the total intensity of probes complementary to the cytosine regardless of its methylation state (U + M). The beta value of a cytosine ranges from 0 (uniformly demethylated state in all DNA molecules present in the sample) to 1 (uniformly methylated state).

For SGS and TGS, instead, the starting data consists of FASTQ and FAST5 files, which contain the nucleotide sequences of the sequenced molecules. In this case, the methylation state of a site is quantified from the fraction of sequences (reads) that support the methylated (unconverted) and unmethylated (converted) state and can be expressed as a fraction of methylated molecules.

With respect to QC, one of the most important controls is the bisulfite conversion efficiency, which for arrays can be performed by analyzing the signal intensity of appropriate control probes, whereas for sequencing, it is performed by examining the conversion efficiency of DNA from genomes with known methylation states (e.g., lambda phage).

For methylation arrays, an additional control is performed on the signal‒to‒noise ratio of the experiment. Usually, samples containing a consistent percentage of probes with a low signal are eliminated, and it is verified that the overall distribution of the slide signal follows the expected bimodal distribution (i.e., with two maxima at 0 and 1).

In the preprocessing phase, cytosines whose methylation estimate is not robust are filtered. In the case of arrays, cytosines that may be affected by nonspecific hybridization with array probes are removed (due to complementarity with multiple regions of the genome or the presence of polymorphisms). In the case of sequencing, sites with very high coverage compared with the average coverage are often removed, as they are affected by duplication artifacts and preferential amplification. In both experiments, sites on sex chromosomes are usually excluded and analyzed separately.

For methylation arrays, data normalization is particularly delicate and aims at removing the background noise of the experiment and removing artifacts generated by the characteristics of the array (dye bias correction and normalization of the signals of type I and type II probes). Furthermore, the evaluation of the batch effect is fundamental, i.e., the systematic differences between samples are not linked to biological characteristics but rather to experimental factors. For this purpose, both unsupervised clustering methods, such as principal component analysis (PCA) and hierarchical clustering (HC), and supervised classification methods, such as exploiting singular value decomposition (SVD) methods of the data, can be used. When identified, the batch effect can be corrected in the preprocessing phase via appropriate algorithms and, in any case, must be considered in subsequent differential methylation analyses.

At this point, one can proceed to identify, typically through the use of linear models, the differentially methylated sites or regions (differentially methylated probes, DMP, or regions, DMR) whose methylation state differs from that of a control cohort, identified taking into account the main covariates that can influence the epigenetic state of a sample (cell lineage of origin, age, sex) as well as the experimental batch. The DMPs/DMRs thus identified can constitute the starting point for association analyses (epigenome-wide association studies (EWASs)) or can be analyzed from a functional point of view through gene-set enrichment techniques (GSEA) to evaluate the involvement of specific groups of manually curated genes (such as MSigDB or KEGG) or can be populated automatically or semiautomatically on the basis of controlled vocabularies (such as Gene Ontologies (GO) or Human Phenotype Ontologies (HPO)). Finally, the differences in methylation levels can be used for the supervised classification of samples via machine learning methods such as support vector machines or random forests.

### Tools for analyzing methylated regions

For the analysis of methylation arrays, multiple open-source tools are available, mostly developed in R language. Among the most commonly used pipelines are minfi [149], methylumi and, among the most recent, SeSame [150], ChAMP [151], and RnBeads [152], which allow the construction of pipelines that cover the entire analysis flow.

With respect to the identification of DMRs, the most widespread algorithms use two alternative strategies:

- **Site-based aggregation:** This group tests phenotypic associations at the level of individual CpG sites and subsequently defines significant genomic regions by aggregating significant sites within a certain distance. This procedure is implemented, for example, in the DMRCate package.
- **Annotation-based regions:** This strategy involves examining predefined genomic regions on the basis of a priori annotations (e.g., promoters and CpG islands) and verifying their associations with a phenotype, calculating a regional p value on the basis of various algorithm-specific functions. The first approach is usually more prone to identifying false positives from a statistical point of view [153].

For GSEA functional enrichment studies, missMethyl [154], methylGO and methylGSA [155] are among the most commonly used methods, as they have been specifically developed considering the data structure of these types of arrays. With respect to machine learning classifiers, the most widely used packages are e1071 and Caret, which allow the use of the main artificial intelligence algorithms, including support vector machines and random forests. Finally, for the analysis of CNVs from methylation arrays, the Conumee package can be used.

The analysis of sequencing data first involves aligning the sequences with the reference genome. Among the available programs, Bismark [156], BSMAP [157], and BS-Seeker2 [158] are commonly used. These programs generate files containing, for each site, the number of reads that support the methylated and unmethylated state. These files can be imported into R for subsequent analyses.

Among the R packages, RnBeads [152] and ChAMP [151] allow adapting functionalities developed for the analysis of methylation arrays to this type of data. In contrast, bsseq [159], DSS [160], and methylKit [161] are among the most commonly used packages developed ad hoc for the analysis of sequencing data.

Finally, some softwares (MethylExtract [162], Bis-SNP [163], BS-SNPer [164] and CGmapTools [165]) allow calling variants from methylation data.

### Key points summary

DNAm is a crucial epigenetic modification, especially in CpG dinucleotides, with diagnostic value in diseases like fragile X syndrome and imprinting disorders. The analysis of DNAm profiles supports diagnosis, particularly for neurodevelopmental disorders and multilocus imprinting disturbances. Experimental methods for DNAm analysis include methylation arrays and bisulfite sequencing, with TGS offering new capabilities for direct methylation detection without bisulfite treatment. Key analysis phases involve quantification, quality control, filtering, normalization, and identifying DMRs. Various bioinformatics tools, like minfi, SeSame, and Bismark, aid in the analysis of DNAm data from arrays or sequencing, supporting functional analysis, CNV detection, and machine learning for classification.

## Conclusions

**Fig. 7:**
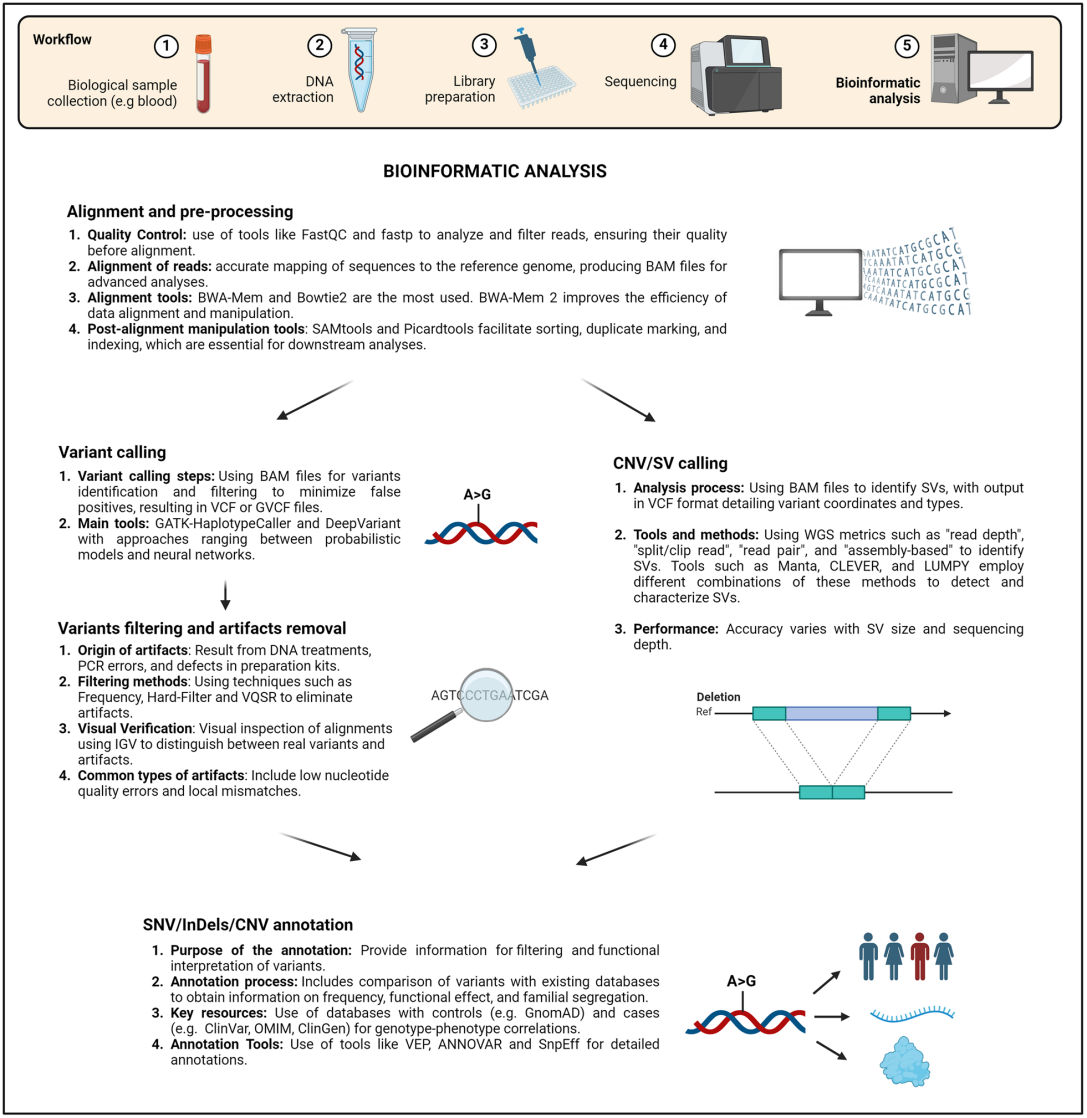
Summary of the variant calling process. Graphical outline of the proposed computational analyses for germline variant calling in short-read sequencing. Created in BioRender. BioRender.com/b98g706

**Fig. 8:**
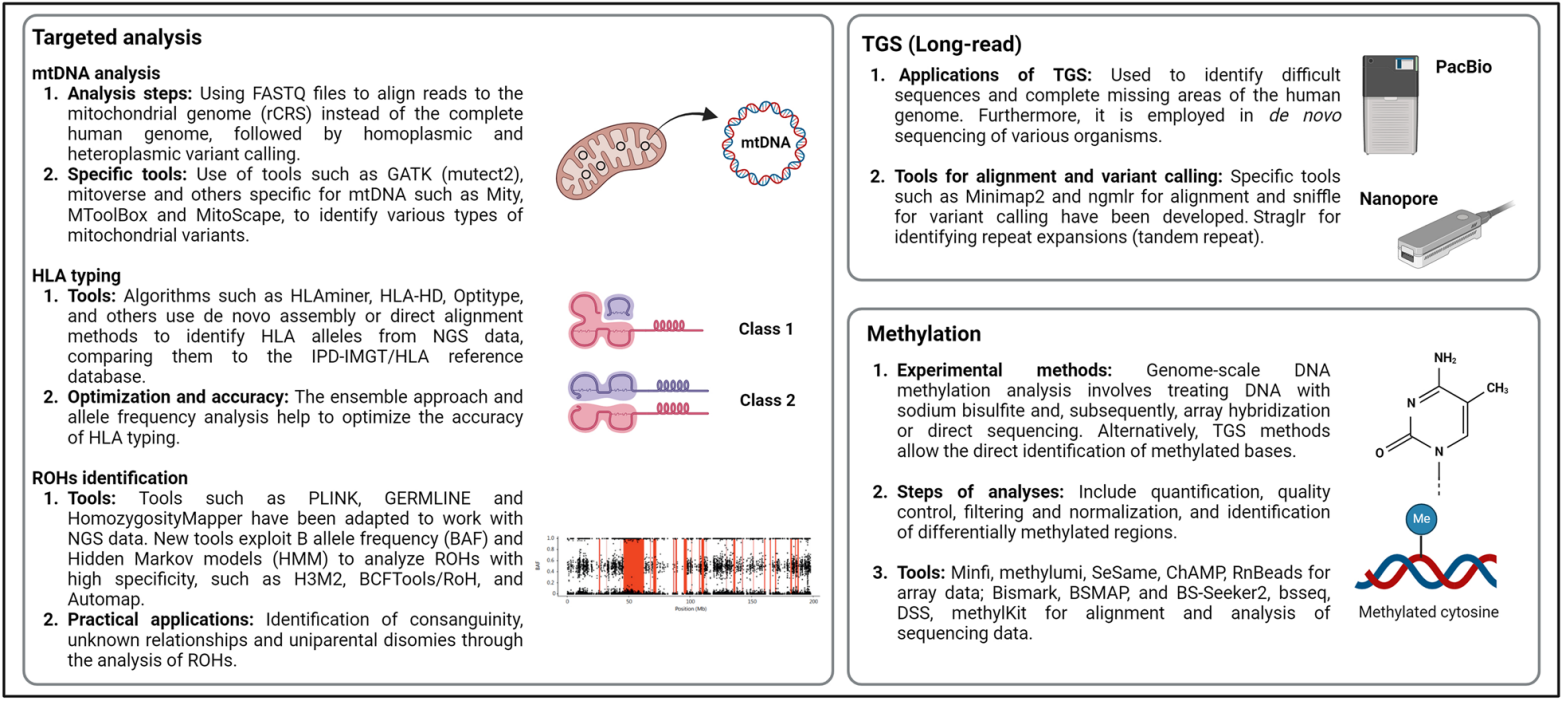
Summary of processes related to targeted analyses, third-generation sequencing, and DNA methylation. Graphical outline describing short-read sequencing targeted approaches, third-generation sequencing based on long reads, and approaches for analyzing genome-wide DNA methylation. Created in BioRender. BioRender.com/b98g706

This document provides best practices for germline variant and DNA methylation analysis using SGS (Fig. 7) and TGS (Fig. 8) data. Advances in sequencing technologies have significantly enhanced our ability to detect, characterize, and interpret genetic and epigenetic variations, revolutionizing human genetics and molecular medicine. The transition from traditional Sanger sequencing to high-throughput SGS and now to long-read TGS has enabled the identification of complex genetic alterations, including structural variants and methylation patterns, which are crucial for understanding the genetic basis of hereditary diseases.

The methodologies and tools discussed herein emphasize the importance of rigorous quality control, accurate alignment, effective variant calling, and comprehensive annotation. Each step in the sequencing data analysis pipeline requires careful consideration to ensure reliability and accuracy in identifying genetic variants. In addition to variant analysis, DNA methylation profiling has emerged as a vital component of the epigenetic landscape, providing valuable insights into the research and molecular diagnosis of various genetic disorders.

Furthermore, it is essential to frame any genotyping process within an effective quality management system that maintains oversight of the entire set of processes carried out in the laboratory. This ensures consistency, traceability, and adherence to quality standards throughout the sequencing workflow, from sample preparation to data interpretation, thereby minimizing errors and improving the overall reliability of results.

The integration of new technologies and approaches, such as machine learning for variant impact prediction and ensemble methods for structural variant calling, highlights the ongoing evolution and improvement in this field. By providing good practices and highlighting the most effective tools and techniques, this document aims to support researchers and clinicians in their efforts to diagnose and manage genetic diseases. The use of SGS and TGS technologies, coupled with robust DNA methylation analysis, not only enhances our understanding of the genetic and epigenetic underpinnings of diseases but also opens new avenues for personalized medicine and targeted therapies.

As sequencing technologies continue to advance, it is imperative to stay updated with the latest developments and adapt best practices accordingly. The insights and recommendations provided in this document are intended to serve as a valuable resource for optimizing genetic and epigenetic analyses, ultimately contributing to better health outcomes and advancing the field of genomics.

## Data Availability

Not applicable.

## Abbreviations

SGS: Second-generation sequencing
TGS: Third-generation sequencing
HLA: Human leukocyte antigen
ROH: Runs of homozygosity
BAF: B Allele frequency
WES: Whole-exome sequencing
WGS: Whole-genome sequencing
QC: Quality control
InDels: Insertions and deletions
SNVs: Single nucleotide variants
SVs: Structural variants
CNV: Copy number variant
PCR: Polymerase chain reaction
VCF: Variant call format
GVCF: Genomic VCF
IGV: Integrative genomics viewer
BCR: Balanced chromosomal rearrangements
bp: Base pairs
Mb: Megabases
RD: Read depth
SR: Split/clip read
RP: Read pair
AB: Assembly based
SNPs: Single-nucleotide polymorphisms
SMRT: Single-molecule real-time sequencing
ZMW: Zero-mode waveguides
DMP: Differentially methylated probes
DMR: Differentially methylated regions
DNAm: Methylated DNA
mtDNA: Mitochondrial DNA
